# Analysis measurements of millet collision coefficient of restitution for mechanized seeding

**DOI:** 10.1371/journal.pone.0320001

**Published:** 2025-05-28

**Authors:** Yifei Li, Wenqi Zhou, Shujuan Yi, Tao Chen, Dongming Zhang, Song Wang

**Affiliations:** 1 College of Engineering, Northeast Agricultural University, Harbin, Heilongjiang, China; 2 College of Engineering, Heilongjiang Bayi Agricultural University, Daqing, Heilongjiang, China; Universitas Airlangga, INDONESIA

## Abstract

The millet collision coefficient of restitution (COR) is one of the important elementary physical parameters for researching the precision hole seeding system. In this study, to explore the factors affecting the bounce issue observed when seeds exit the seed-metering and seed-guiding devices, the dynamic equations for the millet collision process were derived employing Newtonian kinematics, and a COR measurement test bench was built. The effects of collision material type, speed and variety respectively on the COR were investigated by single-factor experiments and orthogonal experiments. Single-factor tests revealed that COR values decreased in the sequence of Acrylic plate, ABS, Rubber, Black soil,Meadow soil, and Sandy loam soil, with Tuogu exhibiting higher COR than Zhang Zagu 13 and Shuangyu, the latter having the lowest value. During two collision phases, the COR progressively decreased as seeding speed increased. Orthogonal tests identified the significant influences of each factor. A combination of bench and simulation tests validated the test results, showing no significant differences and confirming the method’s viability for determining COR. These findings offer a reference for optimizing the design of relevant components within the millet metering system.

## Introduction

Millet stands as the predominant coarse cereal in China, accounting for 80% of the global millet cultivation area. It significantly contributes to the adjustment of the agricultural industry structure and the improvement of dietary patterns [1–3]. Annually, China dedicates approximately 1.4 million hectares to millet cultivation, yielding around 2.8 million tons. Notably, ten provinces and autonomous regions, namely Heilongjiang, Hebei, Shanxi, Inner Mongolia, Shaanxi, Liaoning, Henan, Shandong, Gansu, and Jilin, represent 97% of China’s total millet cultivation area [4,5]. Given their small diameter of about 1–3mm, individual millet grains possess limited soil penetration capability. Thus, simultaneous germination of several seeds at proximate distances is essential, creating a “group soil breaking” phenomenon crucial for millet emergence [6,7]. Currently, predominant sowing methods for millets include broadcasting and drilling, which are challenged by high seed rates, excessive water and fertilizer usage, labor-intensive thinning processes, and do not align with the objectives of green and sustainable agriculture [8–10]. Precision hole sowing, as a method that evenly spaces seeds at multiple points while concentrating them locally, not only capitalizes on the group soil breaking effect to ensure seedling emergence rates but also meets objectives of eliminating thinning, conserving seeds, reducing labor, and improving yields by precisely controlling the quantity and spacing of seeds in each hole [11,12]. Our team’s design of a positive and negative pressure millet hole seeding system addresses these challenges through the application of negative pressure for seed sowing assistance, positive pressure for forced seed feeding, and impurity removal, thereby achieving precise seed distribution. Nevertheless, a gap exists between the seed-metering device and the seed ditch, and as seeds exit the seeder and fall into the seed ditch, they are prone to wind influence, leading to collisions. When a seed guide tube is used, seeds may strike the tube’s interior, compromising landing accuracy and resulting in uneven sowing, which affects the quality of hole formation post-landing [13]. To overcome these issues, our team has innovatively developed a spoon chain seed guide mechanism for small grain millet hole sowing, employing a fully constrained active transport method to ensure accurate seed feeding, stable seed transport, and precise seed placement.

Wang et al. [14] examined the collision coefficient of restitution (COR) through both macroscopic experiments and microscopic analyses, deriving a mathematical model for grain-grain COR based on Stronge’s collision mechanics model and the Newton-Euler dynamic equation. The study analyzed energy loss due to translational and rolling motions during collision, obtaining the energy COR for corn grains via laboratory experiments and DEM simulations. Yu et al. [15] utilized high-speed camera technology to assess the kinematic characteristics of garlic during collisions, noting that the motion was purely translational in the compression stage and both translational and rotational in the rebound stage. A higher rotational angular velocity in garlic correlated with a lower measured COR. Wang et al. [16] used high-speed camera technology to examine how different collision parts affect COR, attributing variations to the differing elastic moduli of corn components. Liu et al. [17] conducted single-factor and orthogonal experiments to identify factors influencing the COR of sunflowers, thereby determining the significant factors affecting COR. Current research, both domestic and international, has provided some cumulative insights into COR, primarily focusing on large grain crops, with limited methodologies that integrate bench and simulation tests for COR assessment.

This study focuses on commonly cultivated millet varieties in Heilongjiang, China. Employing high-speed camera technology and principles of kinematics and impacts, it measures the COR of millet under various collision scenarios, identifying factors that influence the collision recovery coefficients. These findings lay the groundwork for the design optimization of components pertinent to the millet hole sowing system.

## Materials and methods

### Materials and machine structure

#### Materials.

**Fig 1:**
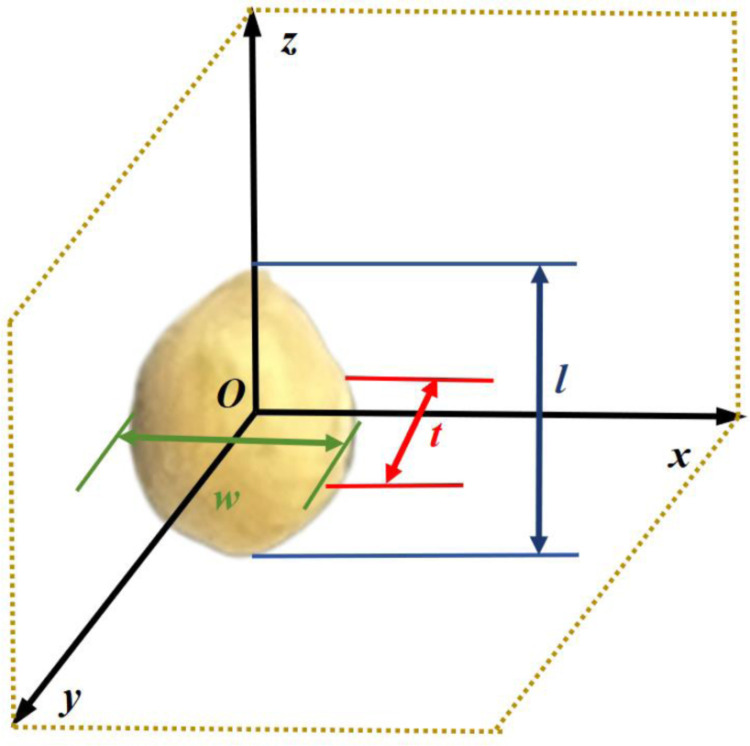
Schematic diagram of the millet characteristic dimensions.

The test materials consisted of Zhang Zagu 13, Tuogu, and Shuangyu, three millet varieties commonly cultivated in Heilongjiang Province, China. Selected in August 2023, the millets were mechanically dried and peeled, yielding seeds with a moisture content of 24–30%. A total of 600 millet seeds were randomly selected as experimental samples, ensuring no external damage or infestation. The seeds were divided into three equal groups for the measurement of their triaxial dimensions: thickness (*t*) as the *x* direction, width (*w*) as the *y* direction, and length (*l*) as the *z* direction. The arrangement is depicted in Fig 1, with measurement results presented in Table 1.

**Table 1:** Appearance parameters of the millet in Heilongjiang.

Parameter	Zhang Zagu 13	Tuogu	Shuangyu
**Length *l*/mm**	1.89~2.11	1.88~2.14	1.86~2.10
**Width *w*/mm**	1.82~1.91	1.72~1.95	1.79~1.85
**Thickness *t*/mm**	1.61~1.75	1.61~1.72	1.69~1.71
**Sphericity *φ*/%**	88.6	86.7	90.2

**Fig 2:**
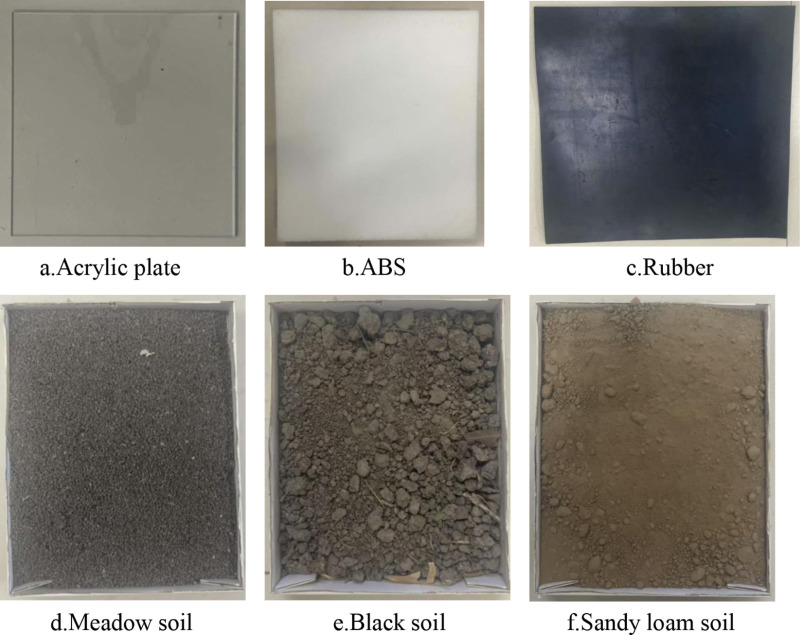
Collision materials.

Six types of collision materials were employed: Acrylic board, ABS, Rubber, Meadow soil, Black soil, and Sandy loam soil, illustrated in Fig 2.

#### Machine structure.

**Fig 3:**
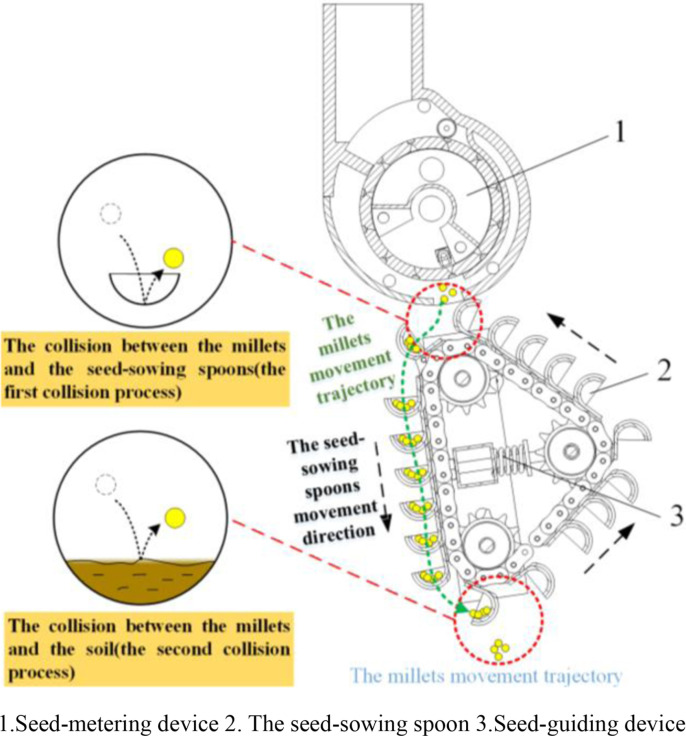
Structure and collision diagram of the millet hole seeding system. 1. Seed-metering device 2. The seed-sowing spoon 3. Seed-guiding device.

The millet hole seeding system includes a positive and negative pressure hole wheel combination seed-metering device and a spoon chain type seed-guiding device, as shown in Fig 3. Utilizing negative pressure for seed filling and positive pressure for forced sowing, the system also incorporates airflow disturbance technology to quantitatively separate millets from seed particles.

After discharge from the seeder, millets enter the seed-sowing spoons of the guiding mechanism, experiencing their first collision. Driven counterclockwise by the device chain, the seed-sowing spoons transport the seeds towards the ground, completing fully constrained seed transportation. As the seed-sowing spoons near the ground, rotation causes the millets to detach due to gravity and inertia, falling into the seed ditch for their second collision, thus concluding the seed-sowing process.

### Test principle

#### Contact model.

The core of collision contact theory involves developing contact mechanics models. This research is grounded in the “elastic-plastic object collision theory model” formulated by Colin Thornton [18,19], employing the energetic COR method to identify factors influencing COR. In sphere collision theory, the contact pressure resulting from two spheres colliding follows Eq (1):


$$P(l)=\frac{3P}{2\pi r^{2}}{\left[1-{\left(\frac{l}{r}\right)}^{2}\right]}^{1/2}$$ (1)

Where *P* is the Contact area pressure with the unit of N, *l* is the horizontal distance from any point on the contact surface to the contact center with the unit of m, *r* is the contact radius with the unit of m.

For collisions between a sphere and a plane, the contact force *P* and the sphere radius *R* within the contact area are detailed in Eqs. (2) and (3), respectively:


$$P=\frac{4}{3}{\mathrm{ER}}^{1/2}\alpha^{3/2}$$ (2)

Where *E* is the elastic modulus of the sphere with the unit of MPa; *α* is the distance between the centroids of two objects with the unit of m; *R* is the radius of the sphere with the unit of m.


$$r={\left(\frac{3\mathrm{PR}}{4E}\right)}^{1/3}$$ (3)

*R* and *α* satisfy the Eq. (4):


$$r^{2}=R\alpha$$ (4)

When yielding occurs during sphere collisions, by integrating Eqs. (2) - (4), Eq. (5) is obtained.


$$\frac{1}{2}mv_{y}^{2}=\int_{0}^{\alpha }P\text{d}\alpha =\frac{8Er_{y}^{5}}{15R^{2}}$$ (5)

Where *v*_*y*_ is the velocity at which the yielding phenomenon of the colliding sphere occurs with the unit of m/s; *r*_*y*_ is the contact radius at which the yielding phenomenon of the colliding sphere occurs with the unit of m; *m* is the effective mass of the spheres with the unit of kg.

When *r* is set to *r*_*y*_, according to Eqs. (1) and (3), it can be obtained that:


$$P_{y}=\frac{2E}{r_{y}}\pi R$$ (6)

Where *P*_*y*_ represents the contact pressure at the moment when the yielding phenomenon of the sphere occurs with the unit of N.

Combining Eqs. (5) and (6),Eq. (7) can be obtained that:


$$v_{y}={\left(\frac{\pi }{2E}\right)}^{2}{\left(\frac{8\pi R^{3}}{15m}\right)}^{1/2}P_{y}^{5/2}=1.56{\left(\frac{P_{y}^{5}}{E^{4}\rho }\right)}^{1/2}$$ (7)

According to references [18,19], it is obtained that:


$$e=1.185{\left(\frac{v_{y}}{v_{i}}\right)}^{1/4}$$ (8)

By substituting Eq. (7) into Eq. (8), it is obtained that:


$$e=1.295{\left(\frac{P_{y}^{5}}{E^{4}\rho }\right)}^{1/8}{\left(v_{i}\right)}^{-1/4}$$ (9)

Where *ρ* Is the material density with the unit of kg/m3; *v*_*i*_ is the velocity of the sphere before collision with the unit of m/s.

According to Eq. (9), the COR is influenced by the material’s elastic modulus, density, and impact velocity. Hence, the experimental variables chosen to determine millet’s COR include variety, collision material, and collision speed.

#### Theoretical analysis.

Collision represents a complex energy transfer process that consistently adheres to the conservation of system momentum and mechanical energy [20]. However, observing the energy conversion process directly is challenging; thus, the changes in velocity before and after seed collisions, as captured by high-speed camera, are utilized to represent the energy conversion situation [21]. This research derives the formula for calculating the COR using the energetic method. Acknowledging that the initial velocity of seeding is not zero during sowing, and based on kinematic principles, the velocity change ratio before and after collision, under a certain initial speed, was determined through collision displacement characterization. The calculation of millet’s COR ensued from this analysis. The specific calculation process is outlined below [17].

In a scenario devoid of external forces, the changes in kinetic energy during the compression and recovery stages of the millets are as follows:


$$\Delta E_{1}=\frac{1}{2}m_{1}(v_{1n}^{2}-v_{11}^{2})+\frac{1}{2}m_{2}(v_{2n}^{2}-v_{22}^{2})$$ (10)

$$\Delta E_{2}=\frac{1}{2}m_{1}(v_{1t}^{2}-v_{11}^{2})+\frac{1}{2}m_{2}(v_{2t}^{2}-v_{22}^{2})$$ (11)

Where ∆*E*_*1*_ is the change in kinetic energy during the compression stage (J), and ∆*E*_*2*_ is the change in kinetic energy during the recovery phase (J*). m*_*1*_ and *m*_*2*_ represent the masses of collision objects 1 and 2, respectively (g), *v*_*1n*_ and *v2n* denote the pre-collision velocities of objects 1 and 2 (m/s), and *v*_*1t*_ and *v*_*2t*_ are the post-collision velocities of objects 1 and 2 (m/s). *v*_*11*_ and *v*_*22*_ signify the instantaneous velocities of objects 1 and 2 at the moment of separation (m/s).

According to the law of conservation of momentum in collision systems:


$$m_{1}v_{1n}+m_{2}v_{2n}=m_{1}v_{1t}+m_{2}v_{2t}$$ (12)

Combining Eqs. (10) - (12), Eq. (13) is obtained.


$$\left\{ \begin{array}{c} \Delta E_{1}=\frac{m_{1}m_{2}{\left(v_{1n}-v_{2n}\right)}^{2}}{2(m_{1}+m_{2})}\\ \Delta E_{2}=\frac{m_{1}m_{2}{\left(v_{2t}-v_{1t}\right)}^{2}}{2(m_{1}+m_{2})} \end{array}\right.$$ (13)

According to the energetic COR definition:


$$e=\sqrt{\frac{\Delta E_{2}}{\Delta E_{1}}}$$ (14)

Where *e* is the coefficient of restitution.

The millets descend freely from the feeding hole at a test bench height *H* with an initial velocity *v*_*a*_, impacting the materials beneath. The grain’s approaching velocity *v*_*0*_ before collision is determined using the kinematic formula:


$$v_{0}^{2}\text{-}v_{a}^{2}=2gH$$ (15)

Where *H* is the falling height of the millets with the unit of mm; *v*_*0*_ is the instantaneous velocity of the grain before collision with the unit of m/s; *v*_*a*_ is the initial velocity of grain falling with the unit of m/s.

The instantaneous velocity of the millets before collision with the collision plates are as follow:


$$v_{0}=\sqrt{2gH+v_{a}^{2}}$$ (16)

Post-collision, a bouncing phenomenon occurs; if air resistance is disregarded, the seeds are influenced solely by gravity, causing their velocity to reduce from the separation velocity *v*_*t*_ to *v*_*0*_. Displacement alterations occur in the *x*, *y*, and *z* directions, denoted as Δ*S*_*x*_ Δ*Sy* Δ*S*_*z*_. In the *x* and *y* directions, the seeds move uniformly, while in the *z* direction, they undergo uniformly decelerated motion.


$$\left\{ \begin{array}{c} \Delta S_{x}=v_{x}\Delta t\\ \Delta S_{y}=v_{y}\Delta t\\ \Delta S_{z}=\frac{1}{2}g\Delta t^{2} \end{array}\right.$$ (17)

Where *∆S*_*x*_ is the displacement change in the *x* direction with the unit of mm; ∆*S*_*y*_ is the displacement change in the *y* direction with the unit of mm; ∆*S*_*z*_ is the displacement change in the *z* direction with the unit of mm; *∆t* is the exercise time with the unit of s.

The velocity *v*_*0*_ subsequent to collision is resolved into components: *v*_*x*_ along the *x* direction, *v*_*y*_ along the *y* direction, and *v*_*z*_ along the *z* direction. This decomposition is described by Eq. (18).


$$\left\{ \begin{array}{c} v_{x}=\frac{\Delta S_{x}}{\sqrt{2g\Delta S_{z}}}\\ v_{y}=\frac{\Delta S_{y}}{\sqrt{2g\Delta S_{z}}}\\ v_{z}=\sqrt[{}]{2g\Delta S_{z}} \end{array}\right.$$ (18)

Because the collision materials are fixed, that is, *v*_*2n*_=*v*_*2t*_=0, so Eq. (19) can be obtained.


$$e=\sqrt{\frac{\Delta E_{2}}{\Delta E_{1}}}=\frac{v_{\text{t}}}{v_{\text{0}}}$$ (19)

Substituting Eqs. (16) and (18) into Eq. (19), the expression for the COR is obtained as follow:


$$e=\frac{v_{t}}{v_{0}}=\frac{\sqrt{v_{x}+v_{y}+v_{z}}}{v_{0}}=\frac{\sqrt{\frac{\Delta S_{x}^{2}}{2g\Delta S_{z}}+\frac{\Delta S_{y}^{2}}{2g\Delta S_{z}}+2g\Delta S_{z}}}{\sqrt[{}]{2gH+v_{a}^{2}}}$$ (20)

Finally:


$$e=\sqrt{\frac{\Delta S_{x}^{2}+\Delta S_{y}^{2}+4g^{2}\Delta S_{z}^{2}}{2g\Delta S_{z}(2gH+v_{a}^{2})}}$$ (21)

By substituting the millets’ displacement changes in the *x*, *y*, and *z* directions into Eq. (21), the COR for millet is determined.

### Test methods

**Fig 4:**
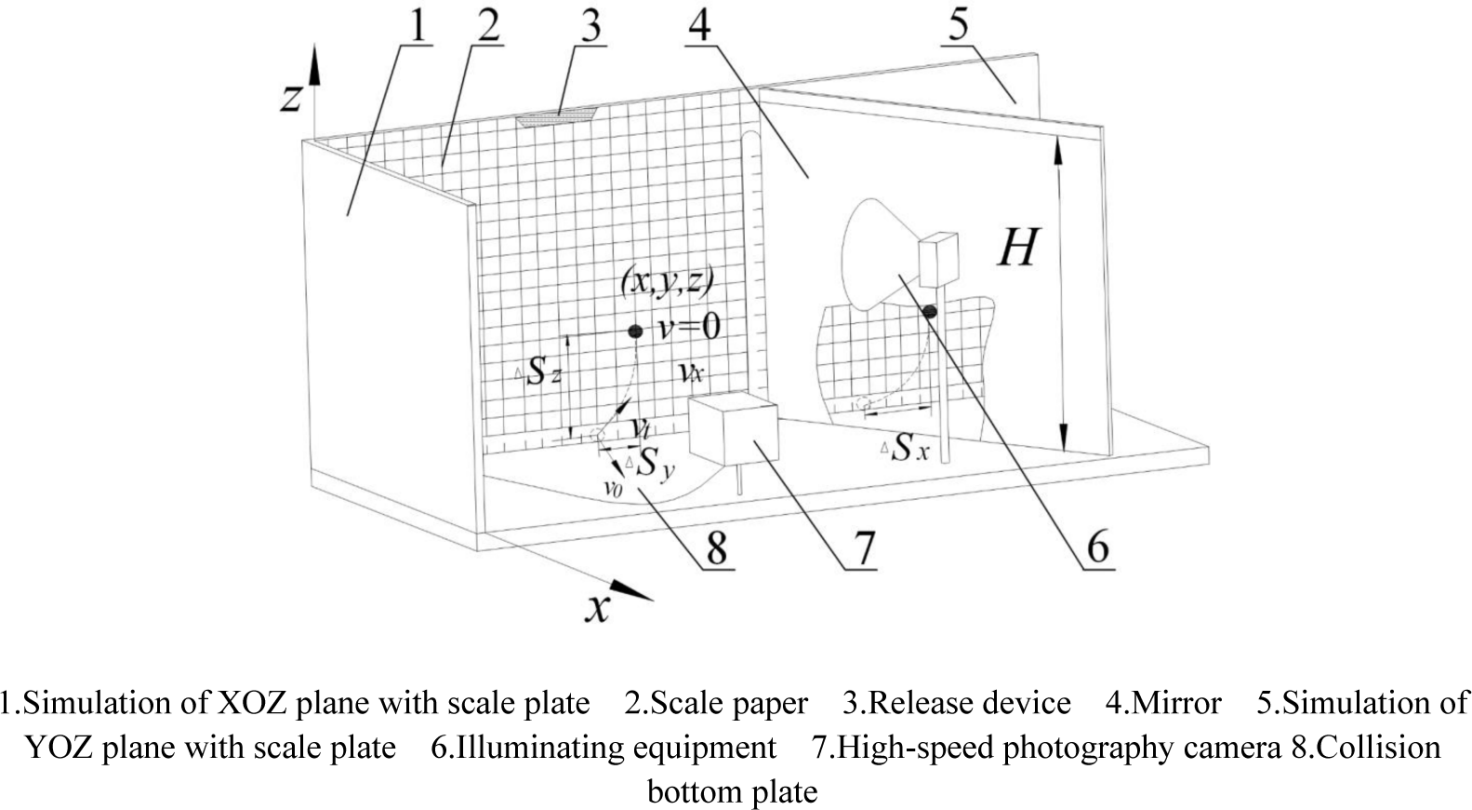
Schematic diagram of analog three-dimensional coordinate system testing device. 1. Simulation of XOZ plane with scale plate 2. Scale paper 3. Release device 4. Mirror 5. Simulation of YOZ plane with scale plate 6. Illuminating equipment 7. High-speed photography camera 8. Collision bottom plate.

The experimental setup comprises material release devices, measuring instruments, camera systems, collision materials, lighting equipment, and a collision floor. Initially, two scale plates are positioned on a horizontal surface at a 90° angle, and a flat mirror is placed at a 45° angle to plate B, set at a specific distance from plate A. Plate A simulates the *xoz* plane, while plate B represents the *yoz* plane. Utilizing the principle of plane reflection, the *x* axis displacement can be directly observed in the plane mirror. The high-speed camera, positioned in front of plate B, and the lighting equipment are carefully adjusted. The configuration of the test bench is illustrated in Fig 4 [16].

**Fig 5:**
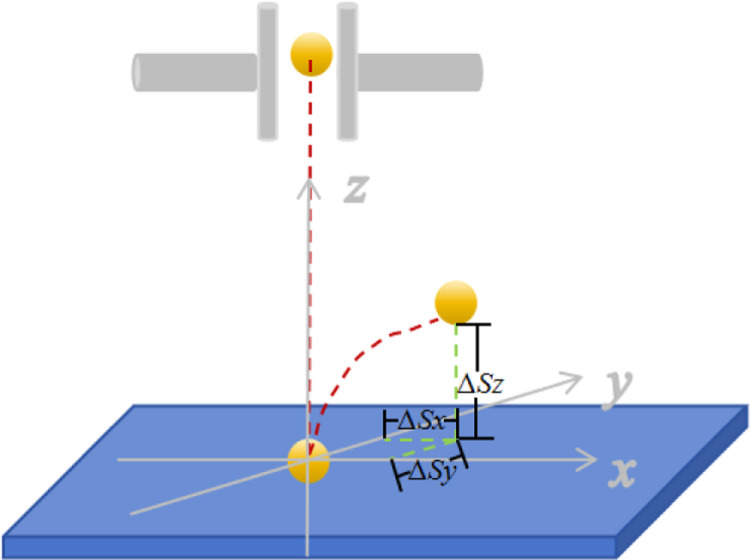
Diagram of displacement variation.

During experiments, the high-speed camera and lighting are activated. Adjusting the drop heights allows for varying initial velocities. The camera, set to 800 frames per second, captures the collision dynamics between millets and various materials. Post-experiment, images depicting the collision point and 0m/s speed are chosen for analysis. The positions of the millets (*x*, *y*, *z*) are designated as the initial location, and the centroid coordinates of the highest rebound point (*x*_*1*_, *y*_*1*_, *z*_*1*_) are recorded. The displacement changes in the three directions are computed to determine the COR [17], with a diagram of displacement variation presented in Fig 5. Each experimental iteration is conducted five times.

### Test scheme

#### Test factors.

The COR of agricultural materials depends on the types of millet, collision materials, and collision speeds. Thus, these variables are selected as the experimental factors. Collision materials, including ABS, rubber, acrylic board, black soil, sandy loam soil, and meadow soil, were chosen based on their common contact with seeds. The guide spoons and soil, which directly interact with the seeds, are among the selected materials.

#### Single-factor test design.

To determine the impact of various factors on the COR, single-factor tests were conducted using millet samples. The COR for different varieties and materials was measured at a planting height of 90mm. For varying speeds, different delivery speeds were applied, and the regression equation determination coefficients were calculated.

Eq. (22) describes the relationship between the linear speed of the seed-metering device and the metering shaft speed. With the metering shaft speed at 30r/min, the seed-metering device’s linear speed is approximately 0.2m/s. Given that the distance from the seeding point to the edge of the seed-sowing spoons is about 1 cm, the speed change is minimal. The speeds of the metering shaft were set at 20, 25, 30, 35, and 40r/min, corresponding to seeding linear speeds of 0.14, 0.18, 0.20, 0.25, and 0.29m/s.


$$v=\frac{2\pi rn}{60}$$ (22)

Where *v* is the seeding linear speed (m/s), *n* is the speed of the metering shaft (r/min), and *r* is the radius of the seed-metering device (mm).

**Fig 6:**
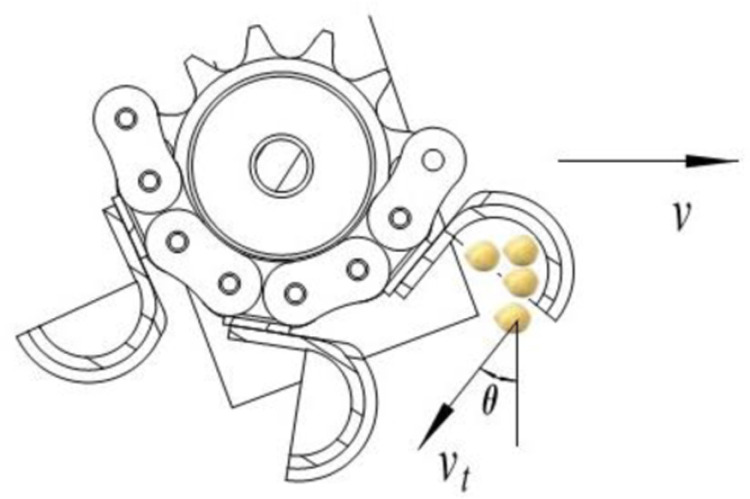
The speed diagram.

To minimize variations in the jumping location post-sowing, efforts were made to achieve zero speed at a seeding angle of approximately 45 degrees. The forward speed of the seeder and seeding speed should fulfill Eq. (22), as illustrated in Fig 6. Typically, seeder forward speeds range from 4.5 to 6km/h, leading to calculated sowing speeds of 1.2m/s, 1.5m/s, 1.75m/s, 2m/s, and 2.25m/s. Each experiment was repeated five times.

#### Orthogonal test design.

To determine the significance and rank of various factors influencing the COR, orthogonal tests were carried out, building upon the single-factor experiments. The factors and their levels are detailed in Table 2(a) and Table 2(b), with each test iteration performed five times for consistency.

**Table 2:** Factors of orthogonal experiment.

Level	Variety	Material type	Seeding linear speeds/(m·s^-1^)
(a) The first collision process			
**1**	Zhang Zagu 13	Acrylic plate	0.18
**2**	Tuogu	Rubber	0.20
**3**	Shuangyu	ABS	0.25
(b) The second collision process			
**Level**	**Variety**	**Material type**	**Sowing speeds/(m·s**^**-1**^)
**1**	Zhang Zagu 13	Meadow soil	1.50
**2**	Tuogu	Black soil	1.75
**3**	Shuangyu	Sandy loam soil	2.00

### Simulation calibration

The Discrete Element Method (DEM) serves as a computational technique to trace the motion of individual particles within a particulate assembly. Preliminary experiments determined the COR of millet under varied conditions. To validate the COR measurements for millet, Zhang Zagu 13 was selected for further study, employing Acrylic plate, Rubber, ABS, Meadow soil, Black soil, and Sandy loam soil as collision materials. This validation combined bench and EDEM simulation tests, verifying the millet collision processes observed in bench tests. The millets’ drop height was set to 90mm, with preprocessing parameters and COR values from bench tests inputted into the software to observe the millets’ movement patterns. Displacement offsets in the *x*, *y*, and *z* directions were recorded, comparing simulated and actual offsets to confirm the COR measurement method’s reliability.

#### Establishment of simulation model.

**Fig 7:**
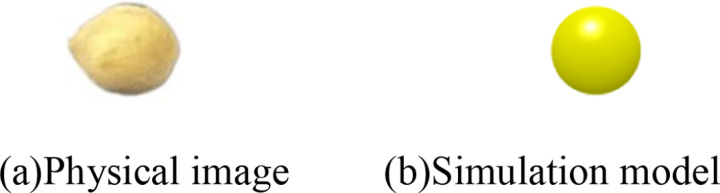
The millet mode. **(a)** Physical image **(b)** Simulation model.

Owing to millets’ high sphericity, seeds were simulated as 2mm diameter spheres, as depicted in Fig 7(a). This simulation model was then integrated into EDEM 2018 software, with the model presentation shown in Fig 7(b).

#### Parameters setting of simulation.

For simulation parameter accuracy, additional measurements were conducted, repeating each experiment five times. The millets’ volume was determined using the volume displacement method, adhering to Archimedes’ principle. This approach led to the calculation of densities for Zhang Zagu No. 13, Tuogu, and Shuangyu as 0.525, 0.619, and 0.482 g/cm3, respectively. These values facilitated the establishment of necessary simulation preprocessing parameters, outlined in Table 3.

**Table 3:** Computational parameters used in the simulations.

Item	Parameter	Value
**Zhang Zagu 13**	Poisson’s ratio	0.38
	Shear modulus (Pa)	5.41*10^7^
	Density (kg⋅m^− 3^)	1170
**ABS plastic**	Poisson’s ratio	0.394
	Shear modulus (Pa)	8.960*10^8^
	Density (kg⋅m^− 3^)	1060
**Rubber**	Poisson’s ratio	0.47
	Shear modulus (Pa)	2.9*10^9^
	Density (kg⋅m^− 3^)	13000
**Acrylic plate**	Poisson’s ratio	0.37
	Shear modulus (Pa)	2.7*10^8^
	Density (kg⋅m^− 3^)	11800
**Meadow soil**	Poisson’s ratio	0.25
	Shear modulus (Pa)	0.79*10^6^
	Density (kg⋅m^− 3^)	1700
**Black soil**	Poisson’s ratio	0.23
	Shear modulus (Pa)	0.72*10^6^
	Density (kg⋅m^− 3^)	2500
**Sandy loam soil**	Poisson’s ratio	0.35
	Shear modulus (Pa)	0.85*10^6^
	Density (kg⋅m^− 3^)	1650
**Corn seed-corn seed**	Static friction coefficient	0.40
	Dynamic friction coefficient	0.021
**Corn seed-ABS plastic**	Static friction coefficient	0.35
	Dynamic friction coefficient	0.033
**Corn seed-rubber**	Static friction coefficient	0.46
	Dynamic friction coefficient	0.038
**Corn seed-polyurethane**	Static friction coefficient	0.34
	Dynamic friction coefficient	0.039

A virtual particle factory was established 90mm above the impact plate, where generated grains fell freely and collided with each other. The total number of generated particles was set to 1, and the total simulation duration was established at 2 seconds. The initial second was allocated for grain generation and descent, with collisions and rebounds concluding after this period, marking the end of the simulation test [22]. Each experimental iteration is conducted fifty times.

## Results and discussion

### Single-factor test analysis

#### Effect of collision variety on COR.

**Fig 8:**
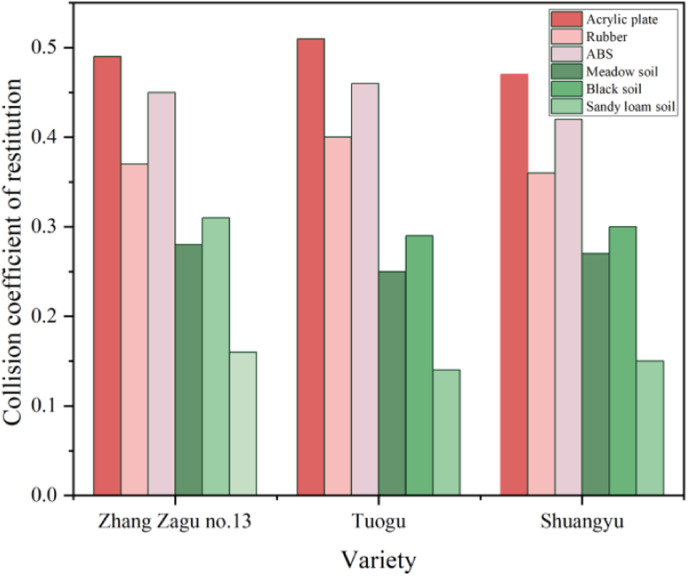
Effect of collision variety on COR.

With a placement height of 90mm, single-factor experiment results for different collision varieties are illustrated in Fig 8. The COR among seeds of various collision varieties is depicted via red color scheme bar charts, while interactions between seeds of different varieties and soil are represented in green. According to Fig 8, the COR values for Tuogu exceed those for Zhang Zagu 13 and Shuangyu, with Shuangyu exhibiting the lowest COR. Gibson et al. [20,23] noted that variations in grain shape, size, and center of gravity affect collision contact areas, rebound angles, and speeds. These factors contribute to differing sliding and rolling displacements, altering rebound paths. Generally, larger particle sizes and reduced sphericity tend to decrease grain rotation speed, minimizing energy loss and yielding higher COR. A correlation exists between size and COR [24].

#### Effect of collision material on COR.

**Fig 9:**
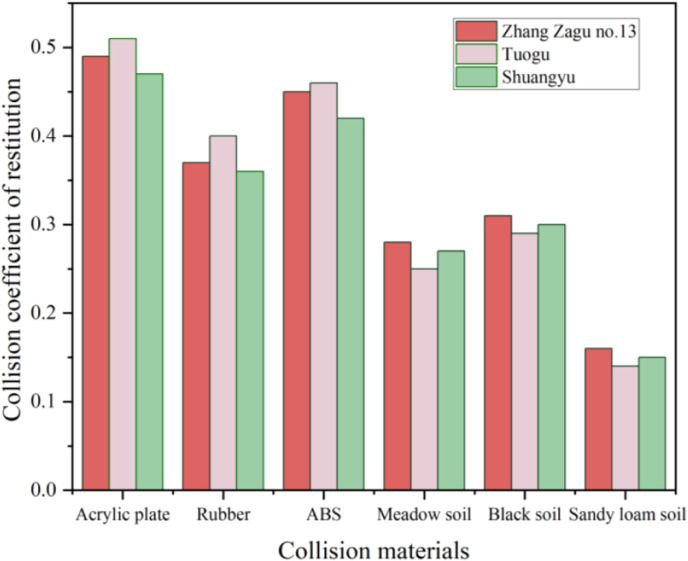
Effect of collision material on COR.

At a placement height of 90mm, single-factor experiments conducted on various collision materials reveal their impact on COR, as shown in Fig 9. The COR values, in descending order, are Acrylic plate, ABS, Rubber, Black soil, Meadow soil, and Sandy loam soil. Specifically, COR values between Black soil and Zhang Zagu 13, Tuogu, and Shuangyu are 0.31, 0.29, and 0.3, respectively. For Meadow soil, the values are 0.28, 0.25, and 0.27, respectively. Sandy loam soil results in COR of 0.16, 0.14, and 0.15, respectively. With Acrylic plates, COR are 0.49, 0.51, and 0.47, respectively. ABS materials yield COR of 0.45, 0.46, and 0.42, respectively, and Rubber shows COR of 0.37, 0.4, and 0.36, respectively. Data analysis indicates the highest COR occurs with millet and Acrylic plate interaction. This outcome is attributed to Acrylic’s high hardness, minimal elastic deformation during collision, and low surface roughness. These characteristics reduce grain rotation and tangential displacement, minimizing friction work and energy loss, hence, a higher COR with Acrylic plates. The COR of ABS materials falls between those of Acrylic plates and Rubber. When millets collide with rubber, the deformation and energy loss are most significant, resulting in the lowest COR. In seed-sowing spoon collisions, the rebound height affects the seeding spoon’s seed distribution efficiency. Excessive rebound may cause seeds to scatter, leading to uneven seed distribution per hole. Considering both mechanical performance and cost-effectiveness, ABS, with its moderate COR and affordability, is recommended as the material for seed-sowing spoons. Additionally, to reduce mechanical damage from impacts on millet, seed-sowing spoon surfaces could benefit from coatings or rubber treatments. Black soil, prevalent in Heilongjiang, China, is characterized by significant compaction and high hardness, leading to the highest COR. In contrast, sandy loam soil, known for its softness and uniformity, exhibits a larger elastic strain energy during collisions, resulting in the lowest COR. Meadow soil, with relatively higher moisture content and less compaction compared to Black soil, has a COR that falls between Black and Sandy loam soils. According to the “recovery coefficient model” by Colin Thornton, a larger elastic modulus generally corresponds to a smaller COR. However, this relationship holds true under specific conditions, such as identical contact pressure, material density, and impact velocity. Given the difficulty in measuring contact pressure accurately, it is imprecise to claim that the elastic modulus solely determines the COR trend [25]. Chitta Sai Sandeep et al. observed that the COR trend between particles and metals differs from that with softer collision materials, like rubber. This insight is crucial for the design of metal components within the seed-metering device, indicating the necessity to consider these factors.

#### Effect of collision speed on COR.

**Fig 10:**
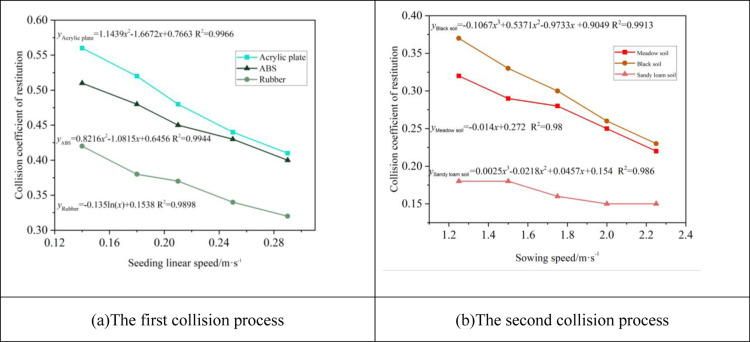
Effect of collision speed on COR. (a) The first collision process. (b) The second collision process.

The effects of collision speeds on COR is illustrated in Fig 10(a) and Fig 10(b). These figures demonstrate a decrease in COR with increasing impact velocities, attributed to the greater compression deformation area of the millet contact area, leading to more energy loss. However, the experimental outcomes are not uniform across all materials due to the distinct kinetic characteristics of the collision materials affecting energy conservation during impact [26,27]. Fig 10(a) depicts the velocity’s impact on Acrylic plates, ABS, and Rubber, with their respective regression equations and determination coefficients (R2) being 0.9966, 0.9944, and 0.9898. Fig 10(b) presents the influence of velocity on Black soil, Meadow soil, and Sandy loam soil, with the regression equations and determination coefficients (R2) documented as 0.9913, 0.98, and 0.986, respectively.

### Orthogonal test analysis

The orthogonal test results for the first collision phase are presented in Table 4, highlighting the significant impact levels and sequences of various factors on the COR: collision material type, variety and seeding linear speed. Notably, the combination of Shuangyu, Rubber, and a seeding linear speed of 0.2m/s resulted in the lowest COR, at 0.36. Conversely, the highest COR, at 0.52, was observed with Zhang Zagu 13, Acrylic plate, and a collision speed of 0.8m/s.

**Table 4:** Scheme and results of orthogonal test.

No.	Variety	Material type		Seeding linear speeds/(m·s^-1^)	COR
**1**	1	1		1	0.52
**2**	1	2		2	0.38
**3**	1	3		3	0.42
**4**	2	1		2	0.50
**5**	2	2		3	0.38
**6**	2	3		1	0.47
**7**	3	1		3	0.45
**8**	3	2		1	0.36
**9**	3	3		2	0.40
***K***_*1*_	1.32	1.47		1.35	
***K***_*2*_	1.35	1.12		1.28	
***K***_*3*_	1.21	1.29		1.25	
*R*	0.11	0.35		0.1	
**Sequence of factors**			Material type>Variety>Seeding linear speed		

Note:*k*_*i*_ is the the sum of levels *i* (*i*=1, 2, 3) in the *j* class of orthogonal tests, and *R* (range) is the difference between max {*K*_1_, *K*_2_, *K*_3_} and min {*K*_1_, *K*_2_, *K*_3_}.

Variance analysis, performed using SPSS software, reflects the significance of experimental factors. According to Table 5, within a 95% confidence interval, material type significantly affected the COR (P<0.01), both variety and seeding linear speeds have a significant impact (P<0.05).

**Table 5:** Analysis of variance in orthogonal test.

Variance source	Sum of squares	DOF	Meansquare	F value	P value	Significance level
**Variety**	0.004	2	0.002	63.000	0.016	*
**Material type**	0.019	2	0.10	289.000	0.003	**
**Seeding linear speeds**	0.002	2	0.001	31.000	0.031	*
**Error**	6.667E-5	2	3.333E-5			
**Sum**	1.690	8				

The orthogonal test results for the second collision phase are detailed in Table 6. These results suggest that the significant levels and order of impact on COR are determined by collision material type, sowing speed, and variety. The lowest COR, at 0.16, was recorded for the combination of Zhang Zagu 13, Sandy loam soil, and a sowing speed of 2.0m/s. The highest COR, at 0.32, was achieved with Tuogu, Black soil, and a collision speed of 1.5m/s.

**Table 6:** Scheme and results of orthogonal test.

No.	Variety		Material type	Sowing Speed/(m·s-1)	COR
**1**	1		1	1	0.28
**2**	1		2	2	0.30
**3**	1		3	3	0.16
**4**	2		1	2	0.25
**5**	2		2	3	0.29
**6**	2		3	1	0.17
**7**	3		1	3	0.26
**8**	3		2	1	0.32
**9**	3		3	2	0.17
***K***_***1***_	0.74		0.79	0.77	
***K***_***2***_	0.71		0.91	0.72	
***K***_***3***_	0.75		0.5	0.71	
***R***	0.04		0.41	0.06	
**Sequence of factors**		Material type>Sowing Speed>Variety			

SPSS software’s variance analysis (Table 7) indicates that within a 95% confidence interval, collision material type has a significantly influence COR (P<0.01), and Variety has a significant impact (P<0.05) with Variety having non-significant impact.

**Table 7:** Analysis of variance in orthogonal test.

Variance source	Sum of squares	DOF	Meansquare	F value	P value	Significance level
**Variety**	0.000	2	0.000	13.000	0.071	
**Material type**	0.300	2	0.015	1333.000	0.001	**
**Sowing speed**	0.001	2	0.000	31.000	0.031	*
**Error**	2.222E-5	2	1.111E-5			
**Sum**	0.568	8				

### Contrast test analysis

**Fig 11:**
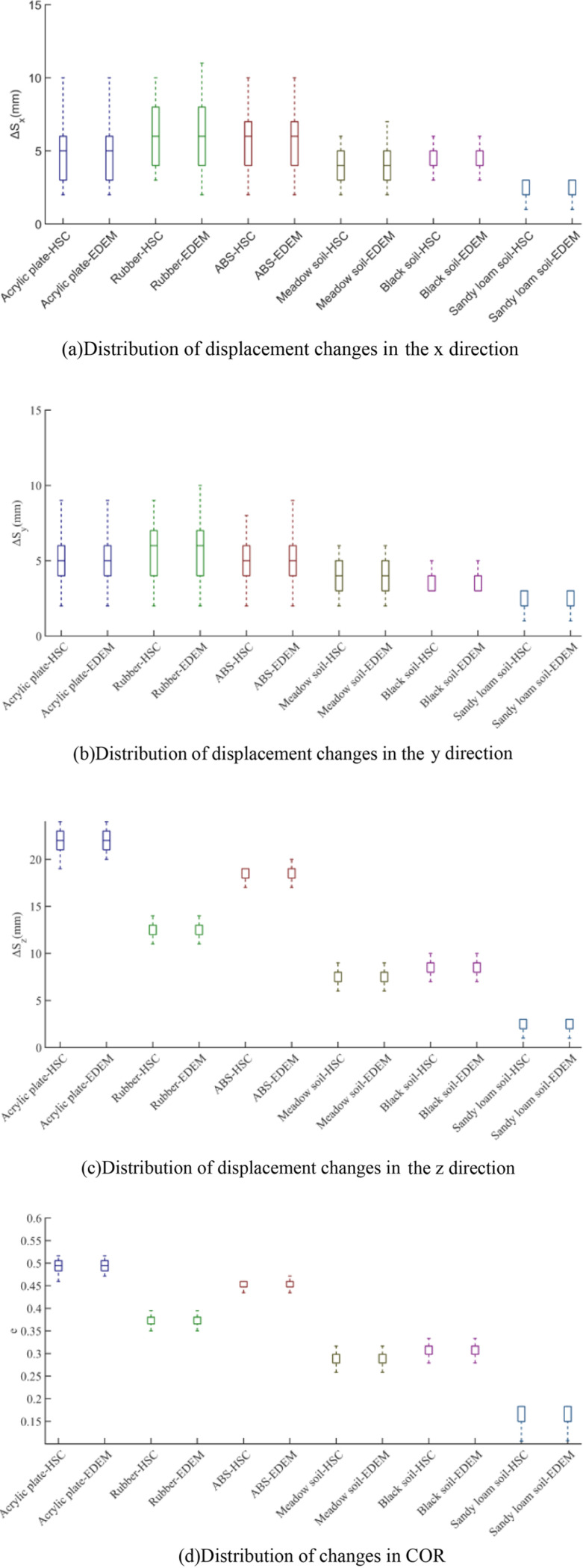
Box plots of the three-dimensional displacement variations and the COR. (a) Distribution of displacement changes in the *x* direction. (b) Distribution of displacement changes in the *y* direction. (c) Distribution of displacement changes in the *z* direction. (d) Distribution of changes in COR.

The article presents the COR distribution information measured by high-speed camera (abbreviated as HSC in Fig 11)and EDEM technology in the form of a box plot, as shown in Fig 11. The two adjacent box lines of the same color in the figure form a comparison group. From the graph, it can be seen that the upper quartile, lower quartile, and median of the data between each group are basically the same, but the upper and lower boundaries are slightly different. The EDEM COR value of rubber in the *x* direction are slightly higher than high-speed camera, and the minimum value are slightly lower than high-speed camera; The EDEM COR in the *y* direction are slightly greater than high-speed camera; The EDEM COR of ABS in the *y* direction are slightly greater than that of high-speed camera, due to the motion behavior of frictional force doing work and elastic-plastic deformation consuming kinetic energy during high-speed camera collisions. However, this article obtains specific differences in displacement changes in various directions through multiple experiments, and found that the COR obtained from the EDEM experiment are slightly higher than that obtained from the high-speed camera experiment, indicating that there is more energy loss during the high-speed camera experiments compared to the EDEM experiments, resulting in a slight difference between the two experiments. However, the differences in displacement changes in all directions don’t exceed 0.5mm.

Among the results of numerous high-speed camera experiments and simulation experiments, groups with the similar collision rebound paths were selected, which were shown in the following text, demonstrating the feasibility and accuracy of the COR measurement method.

In the contrast test illustrations, the calibration line for the collision point is denoted by red centerlines, while the displacement changes in the *z* direction (∆*S*_*z*_), from the collision point to the highest rebound height, are highlighted with yellow lines. Displacement changes in the x-direction (∆*Sx*), from the collision point to the highest rebound height, are marked with red lines. Similarly, displacement changes in the y-direction (∆*S*_*y*_), from the collision point to the highest rebound height, are indicated by orange lines.

**Fig 12:**
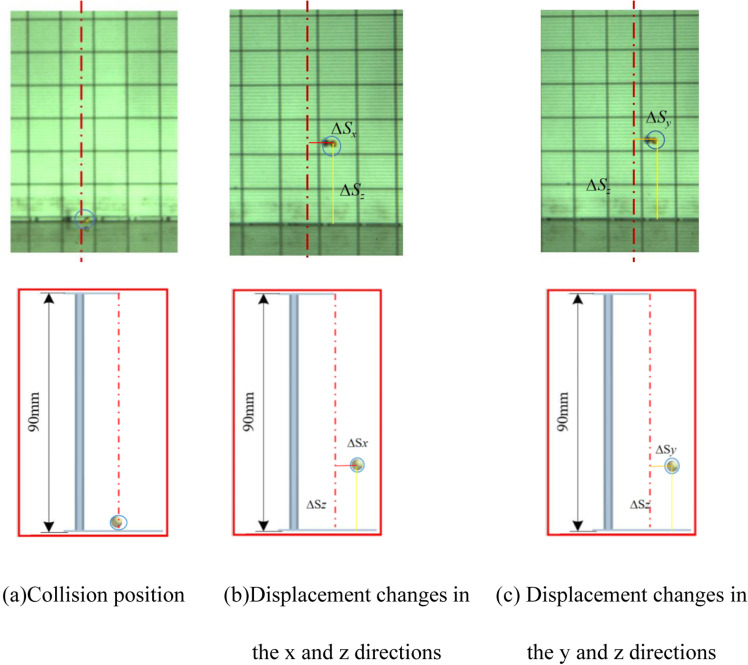
Comparison between simulation tests and bench tests. (a) Collision position. (b) Displacement changes in the *x* and *z* directions. (c) Displacement changes in the *y* and *z* directions.

For collisions between millets and Acrylic plate, Fig 12 demonstrates that the displacement changes in the *x*, *y*, and *z* directions, as captured by the high-speed camera, are 3, 9, and 26mm, with the overall average change being 5.14, 5.08, 21.7, respectively. Consequently, the average COR is calculated to be 0.491. EDEM software measurements for the *x*, *y*, and *z* displacement changes post-collision are approximately 3, 10, and 27mm, with the overall average change being 5.26, 5.36, 21.77, the average COR is calculated to be 0.492, aligning closely with bench test results.

**Fig 13:**
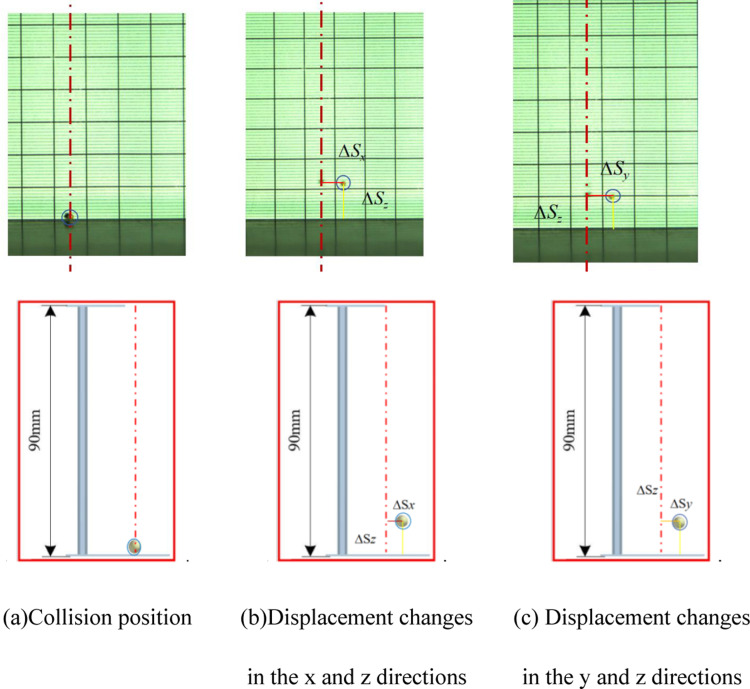
Comparison between simulation tests and bench tests. (a) Collision position. (b) Displacement changes in the *x* and *z* directions. (c) Displacement changes in the *y* and *z* directions.

Regarding collisions with Rubber, as depicted in Fig 13, displacement changes in the *x*, *y*, and *z* directions are 4, 4, and 13mm, with the overall average change being 5.8, 5.68, 12.76, respectively, leading to a average COR of 0.377. Measurements from EDEM software for post-collision displacement changes in *x*, *y*, and *z* are around 2, 2, and 12mm,with the overall average change being 5.98, 5.74, 12.83, the average COR is calculated to be 0.378, showing consistency with bench test outcomes.

**Fig 14:**
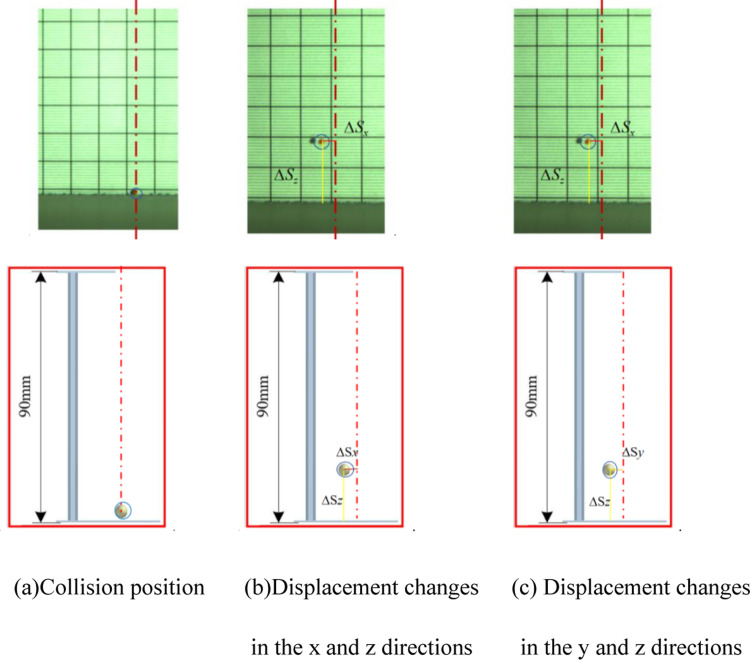
Comparison between simulation tests and bench tests. (a) Collision position. (b) Displacement changes in the *x* and *z* directions. (c) Displacement changes in the *y* and *z* directions.

Collisions between millets and ABS, showed in Fig 14, yield displacement changes in the *x*, *y*, and *z* directions of 2, 2.5, and 20mm, with the overall average change being 5.56, 5.04, 18.16, respectively. This results in a COR of 0.449. Displacement changes in the *x*, *y*, and *z* directions post-collision, measured by EDEM software, closely match the bench test findings at approximately 2, 2.5, and 20mm, with the overall average change being 5.5, 5.36, 18.59, the average COR is calculated to be 0.454

**Fig 15:**
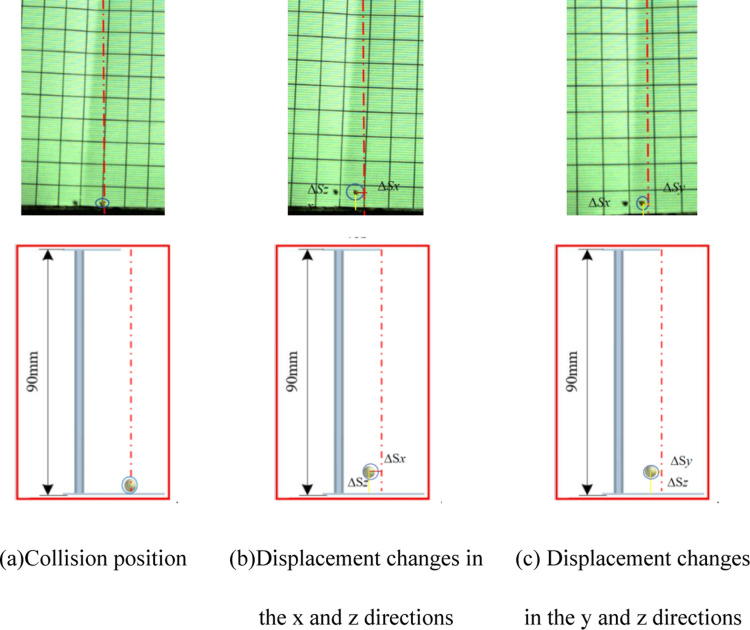
Comparison between simulation tests and bench tests. (a) Collision position. (b) Displacement changes in the *x* and *z* directions. (c) Displacement changes in the *y* and *z* directions.

The displacement changes after collisions between millets and different soil types are illustrated in Fig 15, displacement changes in the *x*, *y*, and *z* directions, as recorded by the high-speed camera, are 2, 1, and 4mm, with the overall average change being 4.12, 3.64, 7.08, respectively. The calculated COR is 0.28. EDEM software’s measurements for post-collision displacements in *x*, *y*, and *z* closely align with bench test results, approximately at 2, 1, and 2mm, with the overall average change being 4.28, 3.98, 7.18, the average COR is calculated to be 0.282

**Fig 16:**
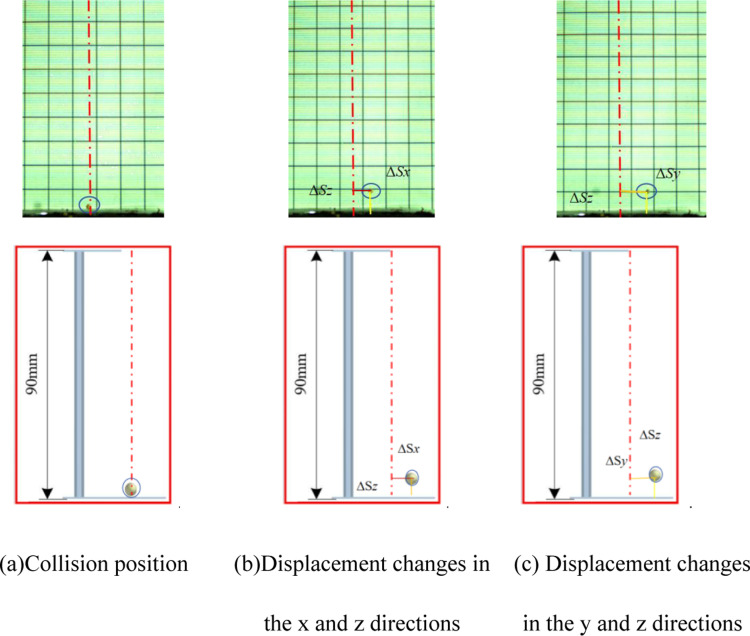
Comparison between simulation tests and bench tests. (a) Collision position. (b) Displacement changes in the *x* and *z* directions. (c) Displacement changes in the *y* and *z* directions.

Regarding collisions with Black soil, depicted in Fig 16, displacement changes in the *x*, *y*, and *z* directions are 6, 8, and 10mm, with the overall average change being 4.24, 3.72, 8.7, respectively. The COR calculated from these displacements is also 0.31. The displacement measurements post-collision by EDEM software, at about 6, 8, and 10mm, with the overall average change being 4.3, 3.78, 8.8, the average COR is calculated to be 0.312, match well with the outcomes of bench tests.

**Fig 17:**
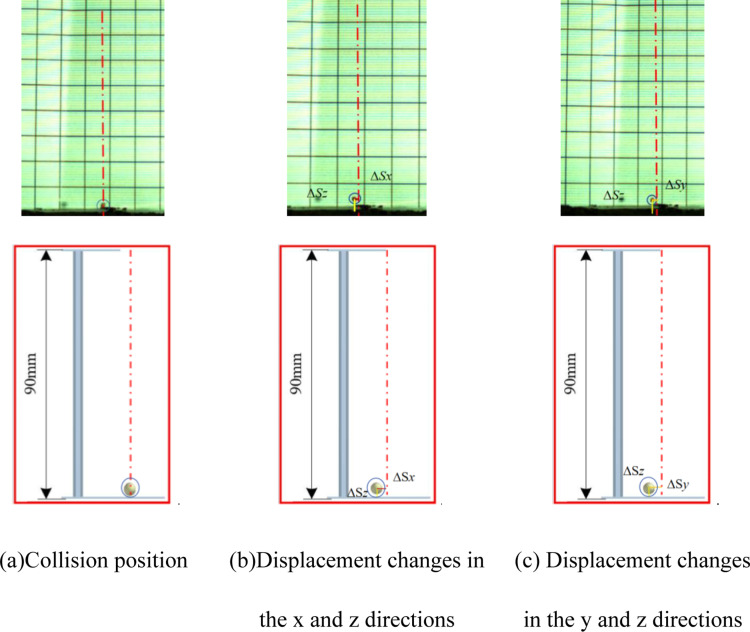
Comparison between simulation tests and bench tests. (a) Collision position. (b) Displacement changes in the *x* and *z* directions. (c) Displacement changes in the *y* and *z* directions.

Collisions with Sandy loam soil, shown in Fig 17, result in displacement changes in the *x*, *y*, and *z* directions of 2, 2, and 2mm, with the overall average change being 2.32, 2.16, 2.3, respectively, leading to a COR of 0.158. Again, these results are in good agreement with the displacement changes measured by EDEM software, which are approximately 2.4, 2.24, and 2.36mm and the average COR is calculated to be 0.16.

The comparison between bench and simulation test results for the collision processes of millets with various soils demonstrates minimal differences in measured displacement changes, highlighting the high reliability of the measured COR for millet. The results indicated that the method for measuring and calculating the COR of millet was feasible, and it also proved that the COR obtained from EDEM simulation experiments could be used for scientific research.

## Conclusions

(1) Based in Colin Thornton’s elastic-plastic object collision theory model, this study identified the principal factors influencing the COR, including collision variety, collision material, and collision speed.
(2) Single-factor experiment results revealed that Tuogu’s COR exceeded those of Zhang Zagu 13 and Shuangyu, with Shuangyu having the lowest value. In collisions with different materials, the COR rankings from highest to lowest were: Acrylic plate, ABS, Rubber, Black soil, Meadow soil, and Sandy loam soil. The COR decreased as collision speed increased.
(3) Multi-factor experiment findings indicated that during the first collision phase, the factors’ significance on COR, in primary to secondary order, were collision material, variety and seeding linear speeds. Material type significantly affected the COR, both variety and seeding linear speeds have a significant impact. During the second collision phase, the factors’ significance on COR, in order, were collision material, sowing speeds, and variety. Collision material significantly affected the COR and sowing speed affected the COR whereas variety also had a notable impact.
(4) The alignment between bench and simulation test outcomes, especially in terms of displacement changes in the *x*, *y*, and *z* directions as captured by high-speed camera tests and EDEM simulation software, confirms the feasibility of this study’s method for accurately determining millet’s COR.

## Supporting information

S1 Data (DOCX)
