# Supplementary material for: Analysis measurements of millet collision coefficient of restitution for mechanized seeding
Source: PLoS One. 2025 May 28;20(5):e0320001. doi: 10.1371/journal.pone.0320001 (PMC12119000; doi:10.1371/journal.pone.0320001)
Supplement: S1 Data — (DOCX) [file pone.0320001.s001.docx]

**Table 1. Appearance parameters of the millet in Heilongjiang**

| **Parameter** | **Zhang Zagu 13** | **Tuogu** | **Shuangyu** |
| --- | --- | --- | --- |
| **Length *l*/mm** | 1.89~2.11 | 1.88~2.14 | 1.86~2.10 |
| **Width *w*/mm** | 1.82~1.91 | 1.72~1.95 | 1.79~1.85 |
| **Thickness *t*/mm** | 1.61~1.75 | 1.61~1.72 | 1.69~1.71 |
| **Sphericity *φ*/%** | 88.6 | 86.7 | 90.2 |

**ZhangZagu13Length:**2.04,1.92,2.05,1.91,1.92,2.03,1.96,2.03,2.05,2.02,2.05,1.94,2.05,2.10,2.08,1.91,1.97,1.97,2.04,2.02,2.06,1.97,1.94,1.91,2.06,1.94,1.98,2.01,1.94,2.03,2.00,1.92,2.06,1.91,1.95,1.94,2.01,1.91,1.98,1.91,1.91,2.06,1.95,2.02,2.10,1.99,2.04,2.06,1.99,2.03,1.91,2.10,1.93,1.95,2.07,2.00,2.06,1.98,1.95,1.90,2.04,1.98,1.99,2.02,1.90,1.96,2.06,2.04,1.92,1.92,1.91,1.89,1.98,2.03,2.05,2.01,1.91,2.03,1.92,1.92,1.91,1.92,1.93,1.93,1.96,1.96,1.94,1.95,2.09,2.04,2.01,1.93,1.94,1.91,2.09,2.05,2.01,1.96,1.93,2.03,2.11,1.93,1.95,1.98,1.91,2.04,1.98,2.11,1.98,2.03,1.92,1.97,1.93,2.06,2.08,1.97,2.04,1.95,2.01,2.07,2.02,1.96,1.96,1.99,1.98,1.97,2.01,2.05,1.98,1.98,1.92,1.90,1.95,1.96,2.03,2.10,2.10,1.99,1.94,2.06,2.06,2.05,2.05,1.91,2.04,1.99,1.94,1.91,2.07,1.93,1.93,2.04,2.09,2.00,2.04,1.92,2.10,2.01,2.04,1.90,2.07,2.05,1.92,2.01,1.96,2.01,1.98,1.98,1.93,1.95,1.89,2.09,2.03,2.10,1.93,2.09,2.06,2.02,1.99,1.95,2.06,1.94,1.90,2.06,2.04,2.05,2.03,1.98,1.98,2.07,1.96,2.07,2.06,2.08,2.00,2.03,2.10,1.99,1.90,2.08

**ZhangZagu13Width:**1.88,1.85,1.91,1.84,1.88,1.87,1.85,1.83,1.82,1.86,1.84,1.89,1.85,1.90,1.89,1.87,1.84,1.91,1.84,1.90,1.84,1.85,1.83,1.88,1.84,1.82,1.89,1.85,1.88,1.85,1.88,1.82,1.90,1.89,1.89,1.89,1.85,1.88,1.87,1.87,1.84,1.84,1.86,1.84,1.89,1.91,1.82,1.87,1.83,1.89,1.91,1.83,1.90,1.82,1.88,1.89,1.87,1.90,1.90,1.88,1.83,1.84,1.84,1.82,1.83,1.88,1.90,1.85,1.86,1.91,1.91,1.88,1.91,1.89,1.85,1.88,1.84,1.85,1.88,1.87,1.86,1.87,1.89,1.87,1.87,1.87,1.87,1.83,1.88,1.91,1.85,1.91,1.85,1.90,1.86,1.86,1.84,1.83,1.85,1.89,1.89,1.88,1.82,1.90,1.90,1.89,1.82,1.85,1.88,1.89,1.84,1.84,1.88,1.86,1.88,1.84,1.84,1.89,1.89,1.90,1.83,1.84,1.83,1.86,1.84,1.90,1.83,1.82,1.87,1.89,1.85,1.84,1.85,1.84,1.87,1.90,1.88,1.83,1.86,1.82,1.87,1.86,1.91,1.89,1.86,1.90,1.83,1.86,1.90,1.90,1.88,1.88,1.85,1.90,1.83,1.89,1.88,1.89,1.86,1.89,1.90,1.85,1.87,1.91,1.87,1.85,1.88,1.85,1.89,1.86,1.86,1.88,1.91,1.85,1.90,1.89,1.91,1.82,1.85,1.88,1.85,1.84,1.88,1.88,1.87,1.88,1.82,1.85,1.86,1.84,1.88,1.90,1.85,1.89,1.83,1.90,1.83,1.87,1.85,1.89

**ZhangZagu13Thickness:**1.68,1.68,1.73,1.66,1.67,1.74,1.62,1.75,1.64,1.70,1.69,1.70,1.66,1.70,1.72,1.61,1.62,1.75,1.70,1.64,1.67,1.63,1.65,1.65,1.66,1.63,1.66,1.63,1.73,1.62,1.74,1.67,1.62,1.66,1.71,1.72,1.69,1.71,1.74,1.62,1.65,1.62,1.64,1.71,1.71,1.73,1.69,1.62,1.74,1.72,1.65,1.69,1.75,1.71,1.73,1.67,1.68,1.69,1.65,1.71,1.68,1.70,1.65,1.63,1.68,1.66,1.72,1.72,1.70,1.63,1.61,1.69,1.65,1.74,1.75,1.65,1.72,1.74,1.69,1.73,1.74,1.69,1.71,1.69,1.61,1.67,1.70,1.68,1.66,1.74,1.73,1.73,1.66,1.69,1.73,1.74,1.70,1.64,1.70,1.62,1.67,1.70,1.74,1.72,1.68,1.72,1.67,1.75,1.75,1.73,1.66,1.67,1.64,1.72,1.73,1.74,1.69,1.69,1.63,1.74,1.67,1.64,1.74,1.72,1.73,1.65,1.70,1.70,1.63,1.67,1.65,1.71,1.65,1.74,1.73,1.66,1.68,1.71,1.73,1.70,1.69,1.66,1.67,1.71,1.73,1.71,1.61,1.70,1.67,1.67,1.63,1.72,1.66,1.64,1.66,1.66,1.69,1.69,1.67,1.67,1.68,1.70,1.74,1.71,1.67,1.73,1.63,1.62,1.62,1.63,1.66,1.65,1.61,1.69,1.62,1.63,1.70,1.73,1.75,1.69,1.75,1.69,1.68,1.66,1.67,1.68,1.62,1.73,1.62,1.67,1.73,1.67,1.70,1.72,1.73,1.74,1.64,1.65,1.74,1.69

**TuoguLength:**1.99,1.90,2.04,2.00,2.06,2.06,2.05,1.89,1.90,1.96,2.02,2.05,1.99,2.09,2.07,2.13,2.02,1.96,1.91,2.04,2.08,1.99,1.90,1.95,1.92,1.95,1.99,2.02,2.00,2.11,2.01,2.13,2.05,2.13,1.94,2.06,1.96,2.05,2.06,1.90,1.95,1.94,2.05,2.10,1.97,2.08,2.06,1.88,2.04,1.98,2.12,1.88,2.00,1.99,2.00,2.08,1.96,2.08,2.00,1.89,1.93,2.07,2.00,1.92,1.97,2.04,1.93,2.07,1.94,2.12,1.95,2.08,1.93,1.95,1.90,2.03,2.06,2.02,1.99,2.05,2.05,2.06,2.05,2.13,1.93,2.06,1.94,1.91,2.04,2.00,2.00,2.05,2.08,1.97,2.05,1.99,2.10,2.10,1.95,2.04,2.03,2.02,2.11,1.95,1.96,1.91,2.12,2.05,2.00,2.05,2.02,2.05,2.02,2.07,2.02,2.14,1.94,1.91,1.91,1.90,1.99,2.00,1.98,2.08,2.04,2.08,2.12,2.13,1.93,1.92,2.06,1.90,2.02,2.02,2.10,2.01,1.98,2.05,2.07,2.02,1.97,1.92,2.03,1.95,1.89,2.08,1.94,2.00,2.06,1.97,2.07,1.98,2.06,2.06,1.99,1.89,1.97,1.99,1.95,1.93,2.09,1.99,2.11,1.98,2.08,1.98,2.09,2.08,1.98,1.94,2.09,2.13,1.97,2.05,1.99,2.10,2.08,1.92,2.10,2.14,2.01,2.11,2.03,1.92,1.93,1.99,2.07,2.09,2.09,1.96,2.02,1.90,1.91,1.92,2.06,2.01,1.93,2.01,1.92,1.89

**TuoguWidth:**1.84,1.86,1.91,1.84,1.77,1.82,1.82,1.94,1.86,1.88,1.89,1.80,1.84,1.85,1.76,1.85,1.88,1.82,1.91,1.89,1.80,1.82,1.81,1.90,1.89,1.82,1.88,1.94,1.90,1.88,1.75,1.81,1.86,1.83,1.73,1.77,1.91,1.72,1.92,1.74,1.87,1.84,1.77,1.85,1.75,1.87,1.86,1.73,1.73,1.76,1.72,1.82,1.91,1.86,1.84,1.92,1.74,1.93,1.74,1.84,1.75,1.85,1.72,1.90,1.92,1.93,1.95,1.84,1.78,1.74,1.84,1.85,1.90,1.74,1.87,1.84,1.76,1.94,1.86,1.82,1.94,1.87,1.82,1.91,1.84,1.85,1.88,1.80,1.78,1.85,1.92,1.81,1.75,1.82,1.79,1.81,1.91,1.81,1.81,1.80,1.75,1.78,1.74,1.82,1.78,1.79,1.82,1.75,1.83,1.88,1.78,1.90,1.74,1.81,1.72,1.77,1.72,1.76,1.75,1.78,1.76,1.75,1.86,1.93,1.94,1.77,1.83,1.81,1.84,1.78,1.74,1.82,1.76,1.73,1.94,1.82,1.94,1.90,1.72,1.88,1.88,1.87,1.85,1.77,1.90,1.77,1.81,1.92,1.92,1.81,1.79,1.86,1.93,1.93,1.86,1.80,1.92,1.82,1.93,1.73,1.84,1.88,1.76,1.80,1.76,1.79,1.81,1.85,1.73,1.85,1.78,1.78,1.78,1.76,1.94,1.94,1.91,1.89,1.76,1.80,1.76,1.72,1.79,1.88,1.86,1.84,1.82,1.79,1.84,1.90,1.90,1.85,1.89,1.87,1.75,1.84,1.80,1.74,1.75,1.77

**TuoguThickness:**1.68,1.66,1.69,1.64,1.61,1.67,1.64,1.71,1.71,1.65,1.61,1.68,1.70,1.72,1.62,1.66,1.67,1.69,1.69,1.68,1.69,1.65,1.67,1.62,1.62,1.72,1.64,1.68,1.72,1.63,1.63,1.65,1.71,1.65,1.64,1.63,1.65,1.65,1.62,1.66,1.62,1.68,1.61,1.67,1.70,1.64,1.66,1.67,1.62,1.66,1.68,1.63,1.70,1.72,1.70,1.67,1.64,1.69,1.64,1.72,1.68,1.68,1.63,1.62,1.64,1.70,1.71,1.69,1.69,1.64,1.67,1.70,1.65,1.72,1.62,1.65,1.67,1.62,1.69,1.67,1.67,1.70,1.70,1.70,1.64,1.66,1.69,1.62,1.62,1.64,1.67,1.72,1.69,1.64,1.64,1.70,1.71,1.68,1.64,1.62,1.70,1.67,1.71,1.62,1.67,1.64,1.70,1.63,1.66,1.65,1.70,1.68,1.63,1.64,1.62,1.68,1.67,1.63,1.63,1.66,1.71,1.67,1.61,1.62,1.70,1.66,1.65,1.70,1.65,1.67,1.69,1.71,1.65,1.68,1.72,1.62,1.67,1.66,1.64,1.64,1.69,1.72,1.63,1.70,1.63,1.72,1.70,1.66,1.69,1.66,1.70,1.65,1.62,1.68,1.71,1.63,1.66,1.69,1.61,1.71,1.69,1.67,1.63,1.66,1.67,1.72,1.70,1.72,1.68,1.65,1.71,1.66,1.64,1.65,1.69,1.67,1.69,1.72,1.72,1.67,1.72,1.62,1.62,1.64,1.67,1.67,1.71,1.67,1.66,1.67,1.69,1.61,1.70,1.63,1.66,1.64,1.65,1.68,1.63,1.64

**ShuangyuLength:**1.91,1.91,1.94,2.07,1.97,1.96,1.90,2.09,1.96,2.06,2.01,1.95,2.07,2.05,1.97,2.06,2.08,1.96,1.94,2.00,2.08,2.03,1.95,2.04,2.09,1.99,1.99,1.93,1.88,1.90,1.88,1.97,1.86,2.08,2.01,1.86,1.87,1.91,1.97,1.89,1.86,2.03,1.94,2.05,1.96,1.96,1.87,1.87,1.88,2.00,1.92,2.06,2.07,2.09,1.98,1.91,1.91,1.99,2.04,1.94,1.97,2.01,2.08,1.90,2.03,2.00,1.96,2.07,1.95,1.90,2.01,2.01,1.94,2.05,2.10,2.10,1.89,1.92,1.87,2.01,1.89,1.96,2.07,1.99,1.95,1.91,1.97,2.09,1.89,1.97,2.07,1.87,2.03,2.09,1.93,1.89,2.02,2.08,2.01,2.08,1.91,2.04,1.94,1.96,1.90,2.06,2.01,2.04,2.05,1.88,2.09,1.98,2.04,2.04,2.06,1.90,1.97,2.01,2.08,2.06,2.07,2.00,2.00,2.07,1.87,2.07,1.96,1.87,2.04,1.90,1.89,2.01,1.92,1.94,1.96,1.96,1.95,2.01,1.90,1.91,1.88,1.94,2.04,1.92,2.04,2.03,2.06,2.06,1.93,1.93,1.99,1.94,2.06,2.05,1.99,1.92,2.02,1.92,1.97,1.95,1.99,2.10,2.04,2.10,1.92,1.99,1.87,2.04,2.00,2.07,2.10,2.08,1.96,1.86,1.99,1.91,1.91,1.94,1.88,2.04,2.04,1.99,1.94,2.06,1.99,2.09,2.07,1.95,1.99,1.94,2.01,2.05,2.04,1.89,2.06,1.87,1.96,2.04,2.05,1.95

**ShuangyuWidth:**1.83,1.84,1.80,1.80,1.80,1.81,1.81,1.85,1.79,1.83,1.80,1.84,1.80,1.82,1.85,1.81,1.79,1.80,1.81,1.81,1.80,1.85,1.83,1.85,1.82,1.85,1.79,1.83,1.84,1.80,1.85,1.84,1.84,1.82,1.84,1.81,1.80,1.81,1.83,1.84,1.81,1.81,1.84,1.83,1.85,1.80,1.84,1.83,1.80,1.82,1.81,1.83,1.84,1.80,1.84,1.84,1.81,1.82,1.82,1.83,1.83,1.84,1.81,1.82,1.85,1.82,1.84,1.81,1.83,1.83,1.85,1.80,1.82,1.82,1.80,1.84,1.84,1.82,1.84,1.80,1.83,1.81,1.82,1.84,1.80,1.81,1.82,1.81,1.84,1.85,1.80,1.80,1.83,1.81,1.85,1.85,1.83,1.84,1.81,1.83,1.85,1.82,1.85,1.83,1.82,1.83,1.84,1.80,1.81,1.85,1.81,1.83,1.84,1.85,1.83,1.80,1.79,1.83,1.82,1.85,1.83,1.83,1.82,1.81,1.85,1.82,1.79,1.83,1.82,1.79,1.84,1.81,1.80,1.80,1.81,1.80,1.83,1.79,1.81,1.81,1.84,1.82,1.84,1.83,1.84,1.80,1.85,1.84,1.79,1.82,1.81,1.83,1.80,1.82,1.83,1.83,1.82,1.79,1.82,1.81,1.80,1.82,1.82,1.82,1.84,1.83,1.84,1.79,1.80,1.82,1.85,1.84,1.82,1.81,1.79,1.83,1.82,1.80,1.82,1.80,1.84,1.81,1.85,1.79,1.84,1.83,1.82,1.83,1.83,1.80,1.84,1.79,1.81,1.80,1.85,1.79,1.82,1.83,1.83,1.84

**ShuangyuThickness:**1.71,1.69,1.69,1.71,1.70,1.69,1.71,1.70,1.70,1.70,1.71,1.71,1.70,1.69,1.70,1.70,1.71,1.70,1.71,1.69,1.69,1.71,1.69,1.70,1.70,1.70,1.70,1.71,1.70,1.70,1.69,1.70,1.70,1.71,1.69,1.70,1.70,1.70,1.69,1.71,1.69,1.70,1.69,1.71,1.70,1.69,1.70,1.70,1.71,1.70,1.71,1.70,1.70,1.70,1.70,1.71,1.70,1.70,1.70,1.70,1.70,1.69,1.71,1.70,1.70,1.69,1.69,1.70,1.70,1.70,1.69,1.71,1.70,1.69,1.71,1.69,1.70,1.70,1.69,1.69,1.71,1.71,1.70,1.69,1.70,1.69,1.70,1.70,1.70,1.71,1.71,1.71,1.69,1.69,1.70,1.70,1.71,1.70,1.70,1.70,1.71,1.70,1.70,1.70,1.70,1.70,1.70,1.71,1.71,1.71,1.71,1.71,1.69,1.69,1.69,1.70,1.69,1.70,1.70,1.70,1.71,1.70,1.70,1.71,1.70,1.69,1.70,1.71,1.70,1.70,1.70,1.70,1.70,1.71,1.69,1.70,1.70,1.70,1.71,1.70,1.70,1.70,1.70,1.71,1.70,1.70,1.70,1.69,1.69,1.69,1.71,1.69,1.71,1.70,1.71,1.71,1.69,1.70,1.71,1.70,1.71,1.70,1.69,1.70,1.69,1.70,1.70,1.70,1.70,1.70,1.69,1.70,1.69,1.70,1.71,1.69,1.69,1.71,1.69,1.69,1.71,1.69,1.71,1.69,1.70,1.70,1.70,1.70,1.70,1.70,1.69,1.71,1.69,1.70,1.71,1.69,1.71,1.69,1.71,1.71

**Fig 8. Effect of collision variety on COR**

| 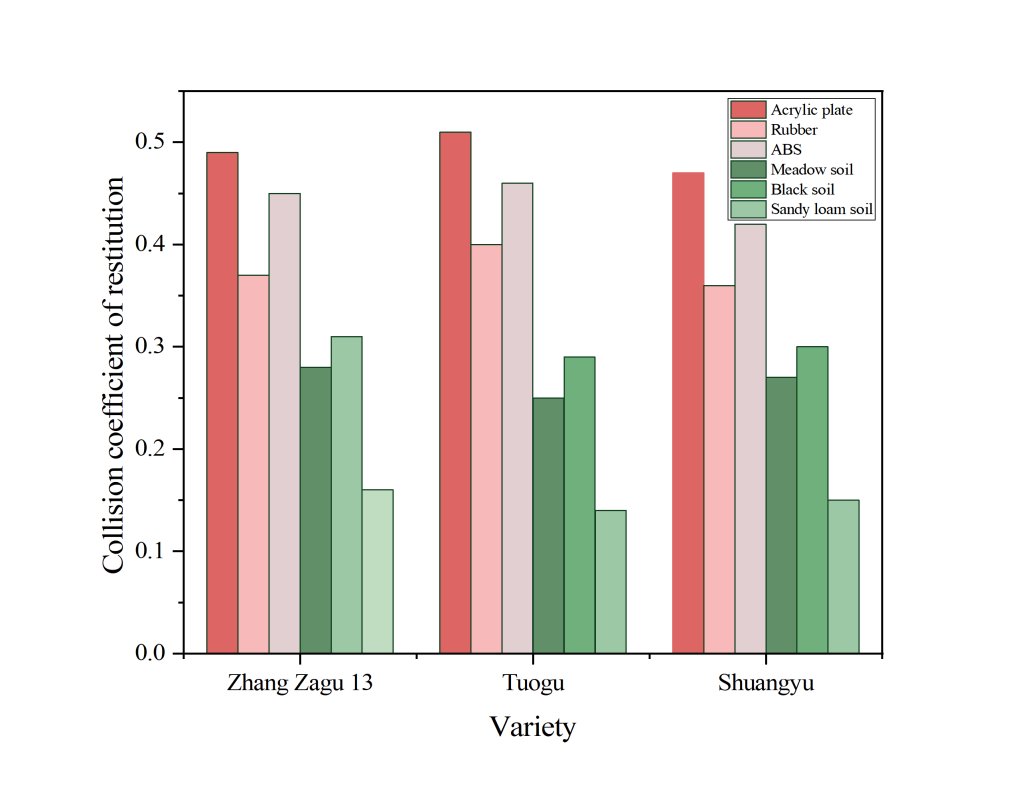 |
| --- |

|  | Acrylic plate | Rubber | ABS | Meadow soil | Black soil | Sandy loam soil |
| --- | --- | --- | --- | --- | --- | --- |
| Zhang Zagu13 | 0.49 | 0.37 | 0.45 | 0.28 | 0.31 | 0.16 |
| Tuogu | 0.51 | 0.4 | 0.46 | 0.25 | 0.29 | 0.14 |
| Shuangyu | 0.47 | 0.36 | 0.42 | 0.27 | 0.3 | 0.15 |

**Fig 9. Effect of collision material on COR**

**
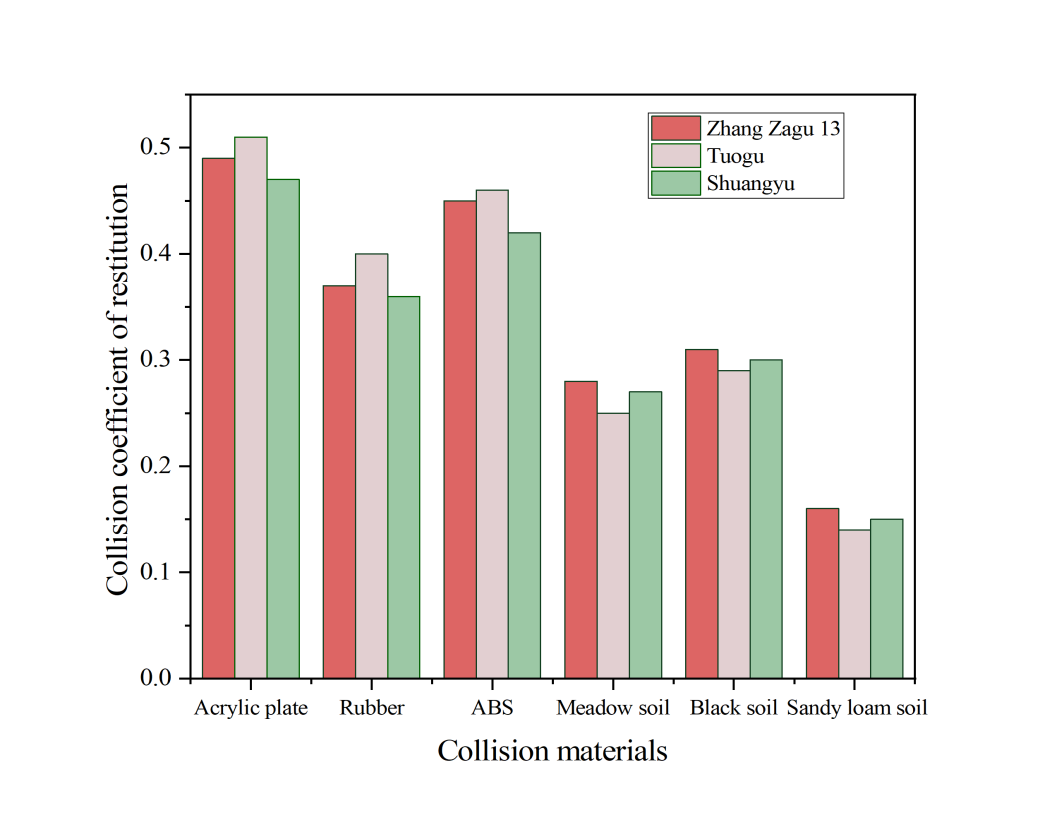
**

|  | Zhang Zagu13 | Tuogu | Shuangyu |
| --- | --- | --- | --- |
| Acrylic plate | 0.49 | 0.51 | 0.47 |
| Rubber | 0.37 | 0.4 | 0.36 |
| ABS | 0.45 | 0.46 | 0.42 |
| Meadow soil | 0.28 | 0.25 | 0.27 |
| Black soil | 0.31 | 0.29 | 0.3 |
| Sandy loam soil | 0.16 | 0.14 | 0.15 |

**Fig 10. Effect of collision speed on COR. (a)The first collision process**


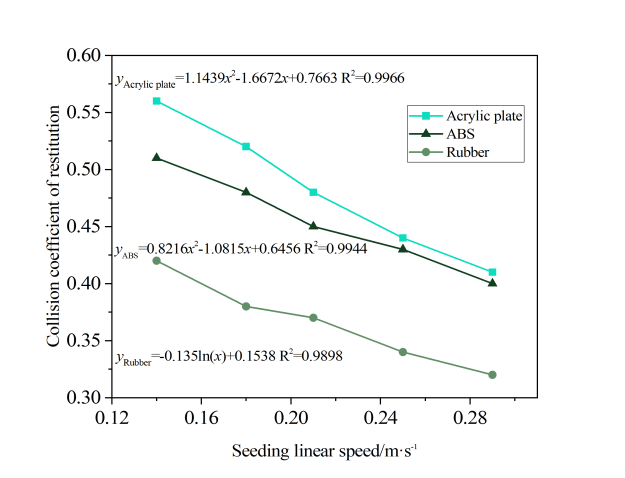


|  | 0.14 | 0.18 | 0.21 | 0.25 | 0.29 |
| --- | --- | --- | --- | --- | --- |
| Acrylic plate | 0.56 | 0.52 | 0.48 | 0.44 | 0.41 |
| ABS | 0.51 | 0.48 | 0.45 | 0.43 | 0.4 |
| Rubber | 0.42 | 0.38 | 0.37 | 0.34 | 0.32 |

**Fig 10. Effect of collision speed on COR. (b)The second collision process**

**
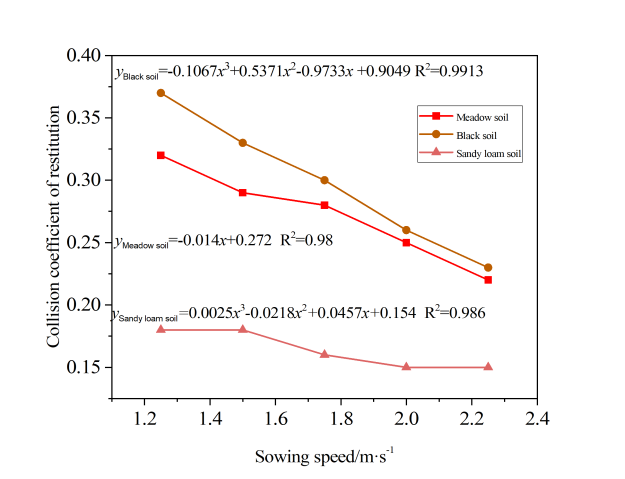
**

|  | 1.25 | 1.5 | 1.75 | 2 | 2.5 |
| --- | --- | --- | --- | --- | --- |
| Meadow soil | 0.32 | 0.29 | 0.28 | 0.25 | 0.22 |
| Black soil | 0.37 | 0.33 | 0.3 | 0.26 | 0.23 |
| Sandy loam soil | 0.18 | 0.18 | 0.16 | 0.15 | 0.15 |

**Fig 11**.**Box plots of the three-dimensional displacement variations and the COR. (a)Distribution of displacement changes in the x direction**


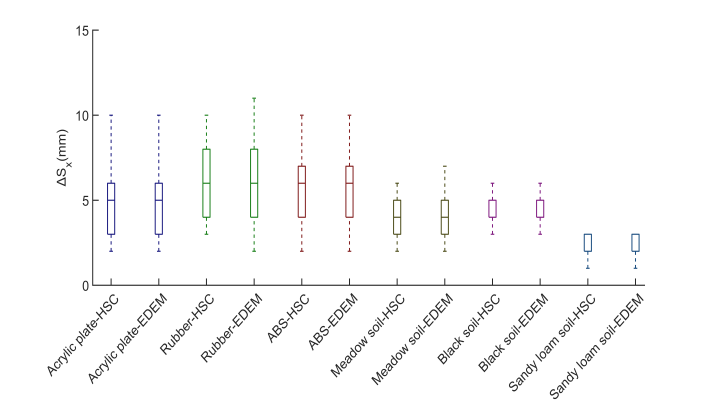


**Acrylicplate-HSC**[4,5,6,4,6,6,6,3,2,6,4,5,3,8,5,4,7,9,6,5,3,4,3,7,3,5,6,4,6,9,5,3,7,5,7,4,3,3,3,5,2,3,10,10,10,6,3,6,4,4]

**Acrylicplate-EDEM[**3.0,3,4,6,4,5,3,4,8,5,7,3,6,3,6,3,6.0,3,10,3,7,3.2,3,5,4,9,6,8,6,5,6,4,3,4,5,5,9,3,2,8,9,3,3,7,9,5,5,6,6,10]

**Rubber-HSC**[4,5,6,3,5,7,4.1,3,4,5,8,9,3.9,10,9,3,4,6,8,9,5,6,4,3,4,8,8,7,10,9,3,7,8,6,4,7,3,6,3,6,5,8,6,3,7,7,3,9,4,5]

**Rubber-EDEM**[3,4,7,6,5,2,8,3,7,11,10,6,3,7,4,4,7,4,11,6,2,4,6,10,6,5,6,6,5,7,10,9,7,8,9,9,6,7,8,8,7,3,7,6,3,11,4,4,3,5]

**ABS-HSC**[2,5,2,10,8,3,2,4,10,10,8,4,6,7,6,6,4,6,4,7,10,8,6,7,6,8,6,7,9,4,3,10,3,5,4,3,4,5,3,2,6,3,6,6,3,7,2,5,8,5]

**ABS--EDEM**[8,7,6,5,2,6,5,8,5,7,6,8,3,6,4,5,5,6,2,6,2,5,5,10,6,3,3,4,7,8,5,7,6,6,7,4,3,2,8,6,2,7,5,8,5,6,7,8,3,7]

**Meadowsoil-HSC**[4,3,5,6,5,4,3,4,3,6,3,6,3,5,6,4,3,5,3,5,3,5,3,3,5,3,4,2,4,2,4,3,4,3,4,3,4,4,4,6,5,5,5,6,3,4,6,4,6,3]

**Meadowsoil-EDEM**[4,5,2,5,4,3,4,6,3,4,5,2,4,3,5,7,4,5,5,5,3,6,6,3,6,2,5,6,4,3,5,4,6,5,6,4,3,3,3,4,5,6,4,4,3,3,4,5-HSC,2,6,]

**Blacksoil-HSC**[5,4,5,3,4,3,5,6,4,3,5,4,6,5,3,5,3,4,4,4,5,4,6,4,4,3,5,3,4,5,4,4,3,4,3,4,3,4,5,3,5,4,4,6,4,5,5,4,6,4]

**Blacksoil-EDEM**[5,4,6,4,5,5,4,5,4,4,3,4,3,4,3,4,4,6,4,5,5,4,6,4,5,5,4,5,4,5,4,5,4,5,4,4,3,4,5,3,5,4,3,5,3,3,4,3,6,5]

S**andyloamsoil-HSC**[2,3,2,3,2,3,2,3,2,3,3,2,2,3,3,2,3,2,3,2,2,3,2,2,3,3,2,3,2,3,2,3,2,3,2,3,2,2,3,2,1,1,2,2,2,2,1,2,1,3]

**Sandyloamsoil--EDEM**[3,2,3,2,2,2,2,3,2,3,2,3,2,3,2,3,2,3,2,3,2,3,2,3,2,3,2,3,2,3,2,3,2,3,2,3,1,3,1,2,3,2,3,3,2,2,1,3,2,3]

**Fig 11**. **Box plots of the three-dimensional displacement variations and the COR. (b)Distribution of displacement changes in the y direction**


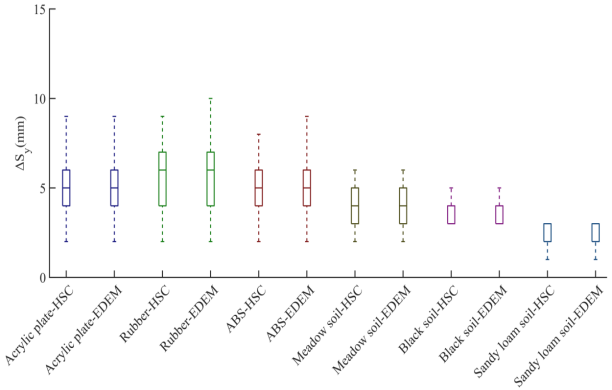


**Acrylicplate-HSC**[7,6,6,4,8,6,4,9,6,4,6,4,3,5,6,3,4,3,3,5,4,3,5,3,4,4,2,4,5,4,2,3,5,3,3,3,5,6,9,4,9,6,6,7,6,8,6,8,7,8,]

**Acrylicplate-EDEM**[6,5,5,6,5,7,5,6,3,4,6,9,7,9,5,6,5,4,3,9,6,9,5,7,5,3,2,6,8,4,8,6,5,4,2,2,4,4,8,4,4,9,6,5,3,9,4,4,3,4,]

**Rubber-HSC**[5,9,3,6,5,7,9,4,4,6,5,6,4,6,4,8,9,6,3,4,3,7,3,7,3,7,8,3,3,7,4,9,9,8,3,8,5,7,4,7,3,5,8,2,6,8,9,4,7,4,]

**Rubber-EDEM**[10,7,9,3,5,9,7,4,5,3,5,6,6,7,5,6,7,8,9,9,7,7,7,3,3.6,7,9,3,4,6,3,7,4,3,5,6,2,7,8,3,5,6,9,9,6,4,7,10,6,7,]

**ABS-HSC**[4,6,6,3,8,6,4,5,6,7,3,5,5,4,4,5,5,4,7,4,5,5,7,2,4,8,4,3,5,6,6,3,6,5,5,3,5,4,6,3,7,5,8,7,4,7,4,2,5,7,]

**ABS--EDEM**[4,6,6,4,9,7,4,5,7,7,3,5,5,4,5,5,5,4,7,6,5,5,7,2,4,8,4,3,6,6,6,4,6,5,5,3,5,4,6,6,7,5,9,7,5,7,4,2,5,9,]

**Meadowsoil-HSC**[4,3,3,5,2,5,4,3,6,3,6,5,5,3,2,4,3,4,4,3,3,4,5,5,5,4,4,4,6,3,5,5,3,4,4,5,2,3,5,3,4,3,4,3,4,5,3,2,4,3,]

**Meadowsoil-EDEM**[4,3,3,6,2,5,4,3,6,3,6,6,5,3,2,4,3,4,4,3,3,4,5,5,5,4,5,4,6,5,5,5,3,4,4,5,2,3,5,3,4,3,4,3,4,5,3,2,4,3,]

**Blacksoil-HSC**[4,3,3,5,4,3,5,3,5,3,5,3,4,3,3,4,4,3,3,3,4,3,4,3,4,3,3,5,4,4,3,5,4,3,3,5,5,3,5,4,5,3,4,4,4,3,3,5,5,3,]

**Blacksoil-EDEM**[4,3,3,5,4,3,5,3,5,3,5,3,4,3,3,4,4,3,3,3,4,3,4,3,4,3,3,5,4,4,3,5,4,3,3,5,5,3,5,4,5,3,4,4,4,3,3,5,5,3,]

**Sandyloamsoil-HSC**[2,2,2,3,1,2,2,3,2,3,1,3,2,1,2,3,2,2,1,2,2,3,1,2,1,2,2,3,2,1,3,1,3,2,3,2,3,2,3,3,2,2,2,3,3,3,2,2,2,2,]

**Sandyloamsoil--EDEM**[2,1,2,2,3,2,2,2,2,2,3,2,2,3,2,2,2,2,3,2,1,2,1,2,2,3,2,3,2,2,3,2,2,3,2,3,2,3,3,2,3,2,3,2,3,2,3,2,1,3,]

**Fig 11**. **Box plots of the three-dimensional displacement variations and the COR. (c)Distribution of displacement changes in the z direction**


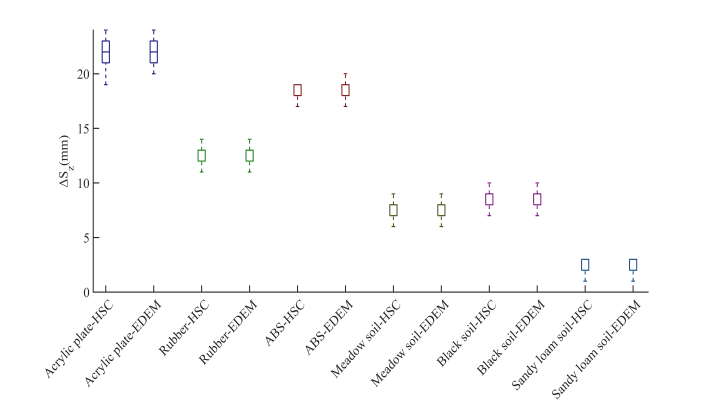


**Acrylicplate-HSC**[21,21,22,23,20,21,24,23,21,23,23,22,23,22,22,21,22,23,22,21,21,23.0,22,22,22,22,21,22,22,21,22,24,21,23,21.7,20.1,21.7,23.1,19,19.9,20.6,21.1,23.2,19,19,21,20.1,21.4,23.2,22.9,]

**Acrylicplate-EDEM**[21,23,22,23,21.0,21,22,21,22,23,21,21,23,21.0,20.2,23,22,22,22,21,23,21,22,21,21,22,20,21,21,22,22,24,21,21,22,20,22,24,21,21,23,23,23,21,20,21,21,22,24,23,]

**Rubber-HSC**[13,12,13,13,12,12,12,14,14,13,13,13,13,11,11,14,13,13,13,11,14,13,13,14,13,13,13,13,13,13,14,13,11,12,13,11,13,13,13,12,13,12,12,13,13,14,13,12,13,13,]

**Rubber-EDEM[**13,14,13,14,12,12,13,14,13,12,12,14,13,13,14,14,12,12,11,13,13,13,13,12,13,13,13,13.5,13,13,13,12,13,13,13,13,14,13,13,12,13,13,12,13,13,11,13,12,13,12,]

**ABS-HSC**[19,18,19,17,18,18,19,18,17,17,18,19,18,18,18,18,19,18,19,19,17,19,18,18,18,18,18,18,18,19.0,18,17,19,18,18,17,19,18,19,19,18,18,18,18,19,18,19,18,17,18,]

**ABS--EDEM**[19,18,18,18,18,18,17,18,19,19,18,18,18,18,19,19,19,19,19,18,19,18,19,19,18,18,19,19,19,19.0,19,18,18,18,18,20,18,20,18,19,19,19,19,19,19,19,19,19,19,18.5,]

**Meadowsoil-HSC**[7,8,7,7,7,9,8,8,8,6,8,6,7,6,7,6,7,6,7,6,7,8,7,7,7,6,6,7,7,6,7,7,7,8,7,6,8,6,7,8,7,6,7,8,7,8,8,8,8,7,]

**Meadowsoil-EDEM**[7,7,8,6,7,7,7,8,7,7,6,8,7,8,8,7,8,7,8,7,8,7,6,6,6,9,7,6,7,6,8,9,7,7,8,7,9,7,6,7,8,7,7,7,7,7,6,7,8,7,]

**Blacksoil-HSC**[10,8,8,9,9,8,9,9,10,7,8,9,8,8,7,8,9,8,9,8,8,10,8,10,8,9,9,8,7,8,8,10,9,8,9,10,9,8,9,10,8,9,10,8,9,10,9,8,10,10,]

**Blacksoil-EDEM**[8,9,8,9,8,8,8,9,8,8,9,10,9,8,9,10,9,9,8,9,10,8,9,10,9,9,9,10,8,7,9,8,9,9,10,9,8,9,7,9,8,9,10,9,9,10,9,10,9,8,]

**Sandyloamsoil-HSC**[2,2,2,2,1,3,2,3,2,2,3,2,1,3,2,3,2,1,2,3,1,3,2,3,2,3,2,3,2,3,2,3,2,3,2,2,2,3,2,3,2,2,3,2,3,2,3,2,2,3,]

**Sandyloamsoil--EDEM**[2,2,3,3,3,2,3,2,3,2,1,2,3,2,2,2,2,3,2,2,3,3,2,2,3,2,3,3,2,1,2,1,2,3,2,2,3,3,3,2,2,3,2,2,3,2,3,3,2,3,]

**Fig 11**. **Box plots of the three-dimensional displacement variations and the COR.(d)Distribution of changes in COR**


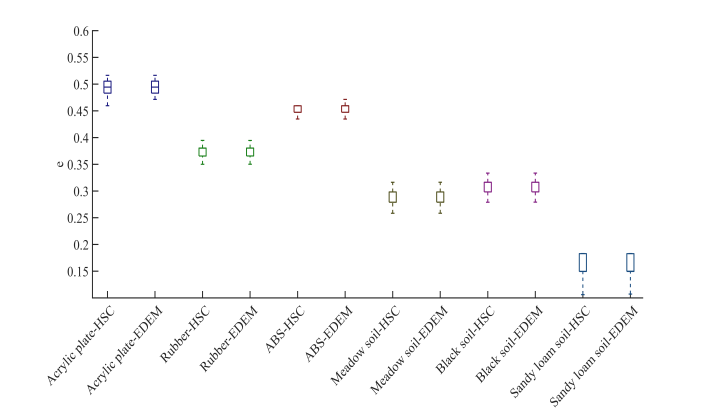


**Acrylicplate-HSC**[0.483138549,0.483132847,0.49450895,0.505564829,0.471557884,0.483148526,0.516458453,0.505636958,0.483102913,0.505589702,0.505589702,0.494467741,0.505547417,0.494531548,0.494494328,0.483081531,0.494499645,0.505636958,0.494473058,0.483117168,0.483081531,0.505556123,0.494458435,0.49449034,0.49444647,0.494467741,0.483102913,0.494455776,0.494494328,0.483184158,0.494451788,0.516418783,0.483151377,0.505567316,0.491109374,0.472619622,0.491076805,0.506678409,0.459617358,0.470287892,0.478548046,0.484258329,0.507885152,0.459715052,0.459693528,0.483188434,0.472650068,0.487763193,0.507798004,0.50452501,]

**Acrylicplate-EDEM**[0.483110041,0.505567316,0.494467741,0.505614574,0.483104339,0.483151377,0.494458435,0.483120019,0.49451028,0.505576022,0.483167055,0.483174181,0.50563074,0.483174181,0.473847852,0.505580997,0.494494328,0.49444647,0.494558131,0.483174181,0.50563074,0.483175949,0.494458435,0.483151377,0.483104339,0.494532877,0.471465872,0.483188434,0.483188434,0.494467741,0.494546169,0.516458453,0.483094361,0.48309151,0.494451788,0.471449001,0.494542181,0.51642695,0.483142825,0.483159929,0.505645662,0.505636958,0.505580997,0.483151377,0.47154255,0.483196985,0.483104339,0.494482364,0.516450286,0.505669288,]

**Rubber-HSC**[0.380178463,0.365498045,0.380190167,0.380166758,0.365313354,0.365471666,0.36547104,0.394457696,0.394457696,0.38023698,0.380318888,0.380400779,0.380149815,0.350114006,0.350204115,0.394596464,0.380342287,0.38026916,0.380272085,0.350204115,0.394494357,0.380307188,0.380131643,0.394557195,0.380111157,0.380389081,0.380432946,0.380228203,0.380579123,0.380438794,0.39447079,0.380438794,0.350042653,0.365478261,0.380131643,0.35002763,0.38015798,0.380307188,0.380131643,0.365428797,0.38015798,0.365260568,0.365478261,0.380096524,0.380307188,0.394701161,0.380321813,0.365468369,0.380213574,0.380178463,]

**Rubber-EDEM**[0.380377383,0.394575521,0.380438794,0.394523159,0.365313354,0.365428797,0.380389081,0.39447079,0.380275011,0.36557717,0.365560687,0.394593846,0.380190167,0.380345212,0.394512685,0.394541486,0.365471666,0.365412307,0.35036175,0.380400779,0.380213574,0.380248682,0.380307188,0.365507937,0.380201754,0.380275011,0.380400779,0.38742278,0.380178463,0.380307188,0.380377383,0.36557717,0.380248682,0.380272085,0.38036861,0.380400779,0.394510067,0.380345212,0.380432946,0.36538922,0.380275011,0.380190167,0.36557717,0.380400779,0.380190167,0.350117761,0.380248682,0.365531016,0.380190167,0.365392519,]

**ABS-HSC**[0.459640541,0.447274672,0.459534549,0.434840484,0.447344721,0.447294431,0.459481544,0.447258506,0.434879609,0.434840484,0.447443489,0.459536206,0.44739321,0.447330353,0.44739321,0.44728545,0.459521298,0.447294431,0.459536206,0.459556081,0.435004783,0.459589204,0.447457853,0.447346517,0.447294431,0.447344721,0.447307004,0.447330353,0.447375252,0.459536206,0.447294431,0.434840484,0.459509704,0.447287246,0.447287246,0.43482679,0.459600797,0.447287246,0.459509704,0.459534549,0.447307004,0.447274672,0.44739321,0.447323169,0.459589204,0.447346517,0.459489826,0.447287246,0.434756355,0.447323169,]

**ABS--EDEM**[0.459600797,0.447366272,0.447342925,0.447287246,0.447366272,0.447366272,0.434693737,0.447373456,0.459590861,0.459630606,0.447294431,0.447373456,0.447274672,0.447307004,0.459536206,0.459551112,0.459551112,0.459554425,0.459556081,0.447342925,0.459516329,0.447303412,0.459590861,0.459640541,0.447307004,0.447344721,0.459509704,0.459509704,0.459609077,0.459633918,0.459569331,0.447330353,0.447342925,0.447323169,0.447346517,0.471442866,0.447274672,0.471435197,0.44739321,0.459587548,0.459556081,0.459590861,0.459643853,0.459655444,0.459551112,0.459609077,0.459575955,0.459580924,0.459524611,0.453606436,]

**Meadowsoil-HSC**[0.279071809,0.298251515,0.27925682,0.279219828,0.27925682,0.316354772,0.298293938,0.298293938,0.298251515,0.258767692,0.298251515,0.25887013,0.279071809,0.258516082,0.279338186,0.258581337,0.279019984,0.258581337,0.279019984,0.258581337,0.279019984,0.298348474,0.279019984,0.279071809,0.279138427,0.258366863,0.258497434,0.27898296,0.279071809,0.25838552,0.279071809,0.27898296,0.279123625,0.298293938,0.279071809,0.258366863,0.298336356,0.258432157,0.279071809,0.298457517,0.27919023,0.258665214,0.279101419,0.298457517,0.279071809,0.298293938,0.298457517,0.298390884,0.298512023,0.279019984,]

**Meadowsoil-EDEM**[0.279123625,0.279138427,0.298221208,0.258767692,0.279034792,0.279138427,0.279123625,0.298415116,0.279219828,0.279071809,0.258767692,0.298384826,0.27919023,0.298251515,0.298318178,0.279367768,0.298293938,0.27919023,0.298390884,0.279138427,0.298251515,0.279271616,0.258767692,0.258516082,0.258767692,0.316329375,0.27925682,0.258683849,0.279271616,0.258516082,0.298445403,0.31643603,0.279219828,0.27919023,0.298457517,0.27919023,0.316293816,0.279019984,0.258516082,0.279071809,0.298390884,0.279219828,0.279123625,0.279071809,0.279071809,0.279138427,0.258432157,0.279101419,0.298263636,0.279219828,]

**Blacksoil-HSC**[0.333511163,0.298293938,0.298318178,0.316456341,0.316390325,0.298251515,0.31643603,0.316456341,0.333511163,0.279019984,0.298390884,0.31643603,0.298457517,0.298390884,0.279071809,0.298348474,0.316354772,0.298293938,0.316390325,0.298293938,0.298390884,0.333441777,0.298457517,0.333472135,0.298293938,0.316319216,0.31643603,0.298251515,0.279123625,0.298445403,0.298293938,0.333441777,0.316319216,0.298336356,0.316319216,0.333472135,0.316319216,0.298336356,0.316400482,0.333441777,0.298445403,0.316390325,0.333441777,0.298457517,0.316354772,0.333511163,0.316481728,0.298336356,0.333528507,0.333511163,]

**Blacksoil-EDEM**[0.298390884,0.316354772,0.298415116,0.31643603,0.298390884,0.298348474,0.298390884,0.316400482,0.298390884,0.298293938,0.316400482,0.333441777,0.316354772,0.298293938,0.316319216,0.333472135,0.316390325,0.316456341,0.298293938,0.316400482,0.333511163,0.298293938,0.316491882,0.333441777,0.31643603,0.316400482,0.316354772,0.333550186,0.298336356,0.27919023,0.316354772,0.298445403,0.316390325,0.316400482,0.333441777,0.31643603,0.298348474,0.316354772,0.27925682,0.316354772,0.298445403,0.316354772,0.333441777,0.31643603,0.316354772,0.333411417,0.316354772,0.333480809,0.316537572,0.298348474,]

**Sandyloamsoil-HSC**[0.149458739,0.149700443,0.149458739,0.149941757,0.10609301,0.182917104,0.149458739,0.183048824,0.149458739,0.149941757,0.182838026,0.149700443,0.106501156,0.182838026,0.149700443,0.182917104,0.149700443,0.106501156,0.149555468,0.182785288,0.106501156,0.183048824,0.149313529,0.182785288,0.149555468,0.182917104,0.149458739,0.183048824,0.149458739,0.182838026,0.149700443,0.182838026,0.149700443,0.182917104,0.149700443,0.149700443,0.149700443,0.182785288,0.149941757,0.182917104,0.149313529,0.149313529,0.182785288,0.149700443,0.182917104,0.149700443,0.182706154,0.149458739,0.149313529,0.182917104,]

**Sandyloamsoil--EDEM**[0.149700443,0.149313529,0.182917104,0.182785288,0.182917104,0.149458739,0.182785288,0.149700443,0.182785288,0.149700443,0.107177945,0.149700443,0.182785288,0.149941757,0.149458739,0.149700443,0.149458739,0.182917104,0.149700443,0.149700443,0.182706154,0.182917104,0.149313529,0.149700443,0.182785288,0.149941757,0.182785288,0.183048824,0.149458739,0.107177945,0.149700443,0.107177945,0.149458739,0.183048824,0.149458739,0.149941757,0.182706154,0.183048824,0.182838026,0.149458739,0.149941757,0.182785288,0.149941757,0.149700443,0.182917104,0.149458739,0.182838026,0.182917104,0.149313529,0.183048824,],,
